# Supplementary material for: Progesterone, Myo-Inositol, Dopamine and Prolactin Present in Follicular Fluid Have Differential Effects on Sperm Motility Subpopulations
Source: Life (Basel). 2021 Nov 17;11(11):1250. doi: 10.3390/life11111250 (PMC8617736; doi:10.3390/life11111250)
Supplement: Supplementary file 1 [file life-11-01250-s001.zip › life-1447561-supplementary.pdf]

Article

Progesterone, Myo-Inositol, Dopamine and Prolactin Present in Follicular Fluid Have Differential Effects on Sperm Motility Subpopulations

Shannen Keyser, Gerhard van der Horst and Liana Maree *

<u>Supplementary Material:</u>

Supplementary Table S1. Comparison of mean SCA motility and kinematic parameters at 5 and 30 minutes for both the low motile (LM) and high motile (HM) sperm subpopulations after treatment with HTF, CAP, HD-C, 1.98 µM progesterone, 3.96 µM progesterone, 19.8 µM progesterone and 11 mM myo-inositol (mean ± SD) (n = 20).

		HTF			CAP			HD-C			1.98 µM PRG			3.96 µM PRG			19.8 µM PRG			11 mM MYO			
		Mean ± SD	SP *p*	TP *p*	Mean ± SD	SP *p*	TP *p*	Mean ± SD	SP *p*	TP *p*	Mean ± SD	SP *p*	TP *p*	Mean ± SD	SP *p*	TP *p*	Mean ± SD	SP *p*	TP *p*	Mean ± SD	SP *p*	TP *p*	ANOVA
**Motile (%)**																							
5 min	HM	66.9 ±21.8***	<0.001	0.552	67.7 ±20.0**	0.009	0.484	66.1 ±20.2**	0.001	0.835	67.1 ±19.0**	0.005	0.633	70.7 ±19.2***	<0.001	0.734	69.9 ±19.9***	<0.001	0.673	74.8 ±15.8***	<0.001	0.233	0.784
	LM	32.5 ±24.2***		0.462	48.6 ±23.1**		0.629	41.9 ±19.3**		0.871	47.0 ±25.5**		0.950	45.1 ±24.7***		0.542	44.1 ±25.6***		0.799	45.6 ±23.8***		0.897	0.558
30 min	HM	62.4 ±19.9***	<0.001		71.8 ±19.2***	<0.001		64.6 ±21.0**	0.002		64.0 ±23.0*	0.018		70.6 ±23.1**	0.006		68.4 ±18.9**	0.002		67.1 ±21.1**	0.005		0.768
	LM	26.8 ±18.5***			44.9 ±27.0***			40.8 ±20.7**			46.5 ±24.5*			49.8 ±25.6**			45.5 ±26.3**			45.9 ±25.6**			0.139
**Prog (%)**																							
5 min	HM	48.8 ±22.1**	0.001	0.585	56.8 ±21.4**	0.001	0.414	54.2 ±21.2**	0.005	0.564	55.8 ±22.0*	0.014	0.734	60.1 ±22.2***	<0.001	0.964	57.8 ±21.3**	0.001	0.669	63.3 ±19.9***	<0.001	0.146	0.524
	LM	21.1 ±21.9**		0.235	34.5 ±22.3**		0.987	32.8 ±19.8**		0.490	35.6 ±26.5*		0.843	35.1 ±23.0***		0.734	32.5 ±24.4**		0.669	34.2 ±22.5***		0.769	0.600
30 min	HM	44.7 ±20.5***	<0.001		61.1 ±21.4***	<0.001		50.0 ±21.2**	0.006		53.3 ±25.0*	0.020		59.8 ±25.4**	0.005		55.2 ±19.7**	0.003		53.8 ±22.4*	0.024		0.332
	LM	12.4 ±11.4*** a			34.4 ±25.2*** b			28.1 ±20.2** b			34.9 ±26.1* b			37.5 ±25.3** b			33.6 ±25.6** b			36.2 ±25.6* b			0.040
**RP (%)**																							
5 min	HM	20.2 ± ab	0.001	0.869	28.2 ± ac	0.004	0.396	17.9 ±9.7* b	0.026	0.564	25.9 ±14.3* abc	0.036	0.285	30.5 ± c	<0.001	0.838	27.6 ± ac	0.001	0.787	31.2 ±16.5** c	0.004	0.771	0.039
	LM	8.0 ±10.7**		0.452	15.2 ±12.4**		0.742	11.6 ±10.0*		0.741	17.4 ±16.5*		0.921	15.4 ±12.4***		0.578	13.7 ±13.2**		0.787	17.8 ±13.4**		0.647	0.293
30 min	HM	20.8 ±10.8***	<0.001		32.2 ±15.7**	0.007		19.5 ±9.8*	0.014		31.2 ±17.3**	0.007		31.4 ±17.1**	0.008		28.8 ±15.8*	0.019		29.7 ±18.4	0.099		0.293
	LM	4.7 ±5.3*** a			18.2 ±15.8** b			12.2 ±12.6* ab			17.0 ±13.6** b			18.2 ±14.3** b			17.5 ±14.8* b			20.6 ±18.2 b			0.027
**MP (%)**																							
5 min	HM	28.7 ±14.0**	0.002	0.286	28.2 ±15.8	0.081	0.482	36.3 ±15.2**	0.003	0.305	29.9 ±14.6*	0.012	0.059	29.7 ±14.2*	0.015	0.753	30.4 ±12.1**	0.005	0.266	32.1 ±14.3*** *	<0.001	0.042	0.675
	LM	13.1 ±12.7**		0.154	19.4 ±11.9		0.352	21.3 ±12.2**		0.170	18.6 ±13.9*		0.854	19.7 ±12.6*		0.927	18.8 ±13.1**		0.266	16.4 ±12.0***		0.972	0.618
30 min	HM	23.8 ±11.0***	<0.001		28.9 ±12.8***	<0.001		30.5 ±16.4**	0.004		22.2 ±11.3	0.259		28.3 ±13.9*	0.029		26.4 ±11.5**	0.005		24.1 ±10.8** *	0.009		0.327
	LM	7.8 ±6.7***			16.2 ±10.4***			15.9 ±10.2**			17.9 ±13.6			19.3 ±12.8*			16.0 ±11.8**			15.5 ±9.7**			0.098
**NP (%)**																							
5 min	HM	18.0 ±11.6	0.054	0.880	10.9 ±5.2	0.132	0.666	11.9 ±6.2	0.152	0.313	11.4 ±6.3	0.549	0.708	10.6 ±5.0	0.462	0.901	12.1 ±8.6	0.888	0.46	11.5 ±7.2	0.652	0.820	0.040
	LM	11.4 ±5.6		0.339	14.0 ±7.0		0.242	9.0 ±5.0		0.262	11.3 ±8.1		0.684	10.1 ±6.7		0.296	11.6 ±8.0		0.651	11.4 ±4.5		0.292	0.354
30 min	HM	17.7 ±11.6	0.327		10.7 ±6.8	0.511		14.6 ±8.2	0.325		10.7 ±5.0	0.633		10.8 ±5.4	0.466		13.2 ±7.8	0.606		13.2 ±11.0	0.319		0.096
	LM	14.3 ±10.4			11.7 ±6.3			12.7 ±8.6			11.6 ±7.2			12.2 ±7.4			12.2 ±7.6			9.7 ±5.8			0.716
**Rapid (%)**																							
5 min	HM	37.2 ±20.9**	0.005	0.977	48.6 ±20.5*	0.002	0.123	50.0 ±21.5**	0.009	0.614	50.0 ±20.6*	0.013	0.827	54.3 ±23.7***	<0.001	0.867	51.7 ±23.0**	0.001	0.714	57.1 ±19.7***	<0.001	0.188	0.164
	LM	17.0 ±20.0**		0.268	28.5 ±20.6*		0.991	30.6 ±19.5**		0.444	31.5 ±25.3*		0.734	31.0 ±21.1***		0.751	28.0 ±23.0**		0.714	29.2 ±20.2***		0.680	0.524
30 min	HM	36.9 ±18.7***	<0.001		56.0 ±22.2***	<0.001		46.7 ±21.3**	0.006		47.9 ±24.0*	0.017		55.6 ±26.0**	0.004		49.3 ±20.0**	0.004		48.9 ±21.7*	0.018		0.180
	LM	9.2 ±9.3*** a			29.4 ±23.4*** b			25.5 ±19.1** b			29.6 ±24.0* b			33.2 ±23.9** b			29.1 ±23.3** b			31.9 ±23.5* b			0.020
**Medium (%)**																							
5 min	HM	16.6 ±10.9**	0.002	0.571	11.4 ±6.3	0.170	0.130	8.6 ±4.8*	0.035	0.927	9.2 ±4.9	0.112	0.938	9.1 ±6.1	0.287	0.380	10.3 ±6.8	0.121	0.796	9.8 ±4.9	0.191	0.503	0.095
	LM	7.0 ±3.9**		0.649	9.2 ±4.4		0.463	5.5 ±3.0*		0.427	7.0 ±4.1		0.784	7.3 ±4.2		0.667	7.5 ±6.0		0.983	8.2 ±5.8		0.897	0.336
30 min	HM	13.5 ±8.4*	0.028		8.8 ±5.1	0.702		8.4 ±4.7	0.262		9.1 ±4.6	0.340		7.2 ±3.3	0.573		10.2 ±6.3	0.146		9.8 ±8.3	0.544		0.073
	LM	6.6 ±5.3*			8.2 ±4.5			6.6 ±4.6			8.3 ±6.9			7.8 ±4.3			7.9 ±5.9			7.6 ±4.8			0.915
**Slow (%)**																							
5 min	HM	13.1 ±8.1	0.220	0.734	7.7 ±3.9*	0.034	0.610	7.5 ±4.3	0.235	0.387	7.9 ±4.7	0.698	0.716	7.3 ±4.0	0.429	0.637	7.9 ±6.3	0.656	0.716	8.0 ±4.9	0.489	0.955	0.196
	LM	8.5 ±4.7		0.359	10.9 ±5.5*		0.115	5.8 ±4.0		0.141	8.5 ±6.7		0.852	6.8 ±5.2		0.163	8.6 ±7.0		0.640	8.2 ±4.0		0.174	0.118
30 min	HM	12.0 ±8.3	0.497		7.0 ±4.4	0.278		9.5 ±5.1	0.325		7.0 ±3.8	0.496		9.3 ±8.9	0.829		8.9 ±6.8	0.981		8.4 ±5.1	0.249		0.251
	LM	10.9 ±9.1			8.5 ±4.4			8.7 ±6.8			8.5 ±5.9			8.8 ±5.6			9.0 ±7.8			6.4 ±4.7			0.575

Supplementary Table S1. Continued…

**Motile VCL (μm/s)**																						
5 min HM	112.7 ±24.5^{a}	0.066	0.692	130.0 ±21.5^{**b}	0.005	0.342	139.3 ±22.6^{b}	0.157	0.238	133.4 ±23.0^{*b}	0.038	0.550	140.2 ±27.9^{*b}	0.043	0.558	137.0 ±25.2^{*b}	0.015	0.318	135.8 ±22.1^{**b}	0.001	0.281	0.018
5 min LM	97.9 ±18.1^{a}		0.105	110.0 ±^{ab}		0.433	127.2 ±26.3^{b}		0.227	116.6 ±28.3^{*ab}		0.980	123.0 ±27.9^{*b}		0.685	117.0 ±27.1^{*ab}		0.558	113.2 ±^{ab}		0.734	0.034
30 min HM	115.7 ±22.0**	0.001		136.6 ±24.7**	0.010		130.6 ±18.8	0.068		129.2 ±23.1	0.122		135.3 ±28.2	0.066		129.5 ±24.1*	0.038		128.0 ±21.2	0.152		0.185
30 min LM	88.1 ±19.4^{**a}			115.9 ±26.0^{**b}			116.9 ±22.9^{b}			116.4 ±30.5^{b}			119.6 ±27.4^{b}			112.0 ±29.7^{*b}			116.0 ±31.1^{b}			0.026
**Motile VAP (μm/s)**																						
5 min HM	61.5 ±13.4**	0.008	0.727	67.5 ±13.2**	0.003	0.395	63.3 ±8.1	0.112	0.983	62.9 ±10.5	0.162	0.437	69.5 ±13.9**	0.002	0.690	67.8 ±12.3**	0.002	0.473	69.4 ±12.2***	<0.001	0.735	0.202
5 min LM	49.6 ±9.5**		0.208	56.1 ±10.7**		0.489	58.4 ±9.5		0.441	57.6 ±12.9		0.949	57.2 ±11.8**		0.892	55.7 ±11.7**		0.824	56.8 ±12.0***		0.548	0.384
30 min HM	59.9 ±12.3**	0.002		70.7 ±12.5**	0.002		63.3 ±7.8	0.024		66.9 ±13.2*	0.031		67.8 ±14.4*	0.019		65.0 ±13.5*	0.044		68.1 ±12.9	0.066		0.201
30 min LM	46.5 ±10.6**			58.1 ±12.6**			55.8 ±10.2			57.9 ±13.7*			57.7 ±13.3*			56.5 ±13.5*			59.5 ±16.9			0.120
**Motile VSL (μm/s)**																						
5 min HM	47.6 ±13.3	0.006	0.763	53.5 ±14.8**	0.005	0.689	44.4 ±10.7	0.183	0.834	47.6 ±11.1	0.263	0.166	53.9 ±13.2**	0.002	0.606	51.8 ±11.7**	0.002	0.645	53.1 ±12.6*	0.014	0.945	0.127
5 min LM	35.0 ±9.8		0.288	42.5 ±10.2**		0.625	39.7 ±9.1		0.939	43.3 ±13.4		0.799	41.4 ±12.4**		0.671	40.3 ±11.2**		0.527	43.3 ±13.5*		0.504	0.386
30 min HM	45.6 ±10.5**	0.002		55.2 ±12.6*	0.014		43.7 ±6.4	0.184		52.9 ±13.6*	0.010		51.9 ±12.9*	0.030		50.1 ±12.6	0.071		53.4 ±13.9	0.154		0.052
30 min LM	32.4 ±11.6**			44.0 ±12.8*			39.5 ±10.9			42.4 ±12.9*			43.1 ±13.5*			42.7 ±13.7			46.5 ±17.4			0.090
**Motile STR (%)**																						
5 min HM	73.7 ±10.4^{*a}	0.041	0.431	75.8 ±9.8^{a}	0.166	0.746	65.9 ±7.6^{b}	0.867	0.938	71.4 ±8.4^{a}	0.823	0.215	74.8 ±7.5^{*a}	0.046	0.486	73.8 ±7.1^{*a}	0.029	0.828	73.3 ±8.4^{a}	0.767	0.756	0.014
5 min LM	65.9 ±9.7*		0.452	72.1 ±8.3		0.609	65.5 ±7.2		0.842	69.6 ±11.9		0.668	69.2 ±10.7*		0.735	68.4 ±8.6*		0.439	71.4 ±11.4		0.549	0.310
30 min HM	71.0 ±8.3^{*ab}	0.041		75.0 ±7.3^{a}	0.090		66.1 ±6.5^{b}	0.986		74.8 ±8.3^{a}	0.051		73.3 ±7.1^{a}	0.221		73.4 ±5.8^{a}	0.305		74.1 ±7.9^{a}	0.771		0.007
30 min LM	64.3 ±9.6*			70.8 ±8.8			66.1 ±12.1			69.5 ±9.4			70.2 ±9.4			70.7 ±10.6			71.6 ±12.4			0.311
**Motile LIN (%)**																						
5 min HM	45.0 ±10.1^{*a}	0.015	0.103	43.3 ±10.9^{a}	0.412	0.611	32.5 ±6.4^{b}	0.819	0.449	36.8 ±7.7^{bc}	0.570	0.065	40.5 ±8.2^{*ac}	0.046	0.531	39.5 ±6.6^{ac}	0.075	0.968	40.4 ±8.3^{ac}	0.553	0.436	< 0.001
5 min LM	36.6 ±7.5^{*ab}		0.722	40.9 ±8.5^{a}		0.467	32.0 ±6.4^{b}		0.409	38.2 ±7.6^{ab}		0.697	35.4 ±8.4^{*ab}		0.525	36.0 ±6.2^{ab}		0.157	38.9 ±9.0^{ab}		0.784	0.018
30 min HM	39.8 ±6.9^{ab}	0.163		41.8 ±8.2^{a}	0.262		34.0 ±4.8^{b}	0.973		41.1 ±7.0^{a}	0.065		39.4 ±6.9^{a}	0.312		39.4 ±4.4^{a}	0.710		42.3 ±7.7^{a}	0.297		0.010
30 min LM	36.7 ±6.9			39.2 ±6.9			34.1 ±8.3			37.4 ±6.1			36.9 ±6.8			38.8 ±6.7			39.6 ±8.8			0.252
**Motile WOB (%)**																						
5 min HM	56.8 ±7.5^{a}	0.102	0.123	54.2 ±8.4^{ac}	0.819	0.847	47.5 ±4.3^{b}	0.649	0.083	50.2 ±4.7^{bc}	0.333	0.061	52.5 ±6.8^{ac}	0.095	0.892	51.6 ±4.7^{c}	0.437	0.970	52.7 ±5.8^{ac}	0.633	0.244	0.004
5 min LM	52.9 ±5.2^{ab}		0.527	53.7 ±6.4^{a}		0.574	48.2 ±4.0^{b}		0.339	51.6 ±4.6^{ab}		0.804	49.2 ±6.2^{ab}		0.395	50.6 ±4.0^{ab}		0.089	51.8 ±6.3^{ab}		0.580	0.022
30 min HM	53.4 ±4.2	0.497		53.7 ±5.9	0.524		49.9 ±3.1	0.862		53.2 ±4.6	0.650		52.3 ±4.7	0.228		51.6 ±2.8	0.275		54.6 ±5.0	0.211		0.061
30 min LM	54.7 ±3.9^{a}			52.7 ±4.6^{ab}			49.6 ±4.7^{b}			51.9 ±4.3^{ab}			50.6 ±4.4^{ab}			52.8 ±4.5^{ab}			52.8 ±4.4^{ab}			0.024
**Motile ALH (μm)**																						
5 min HM	2.9 ±0.7^{a}	0.572	0.201	3.5 ±0.7^{b}	0.064	0.219	4.2 ±0.8^{b}	0.444	0.253	3.9 ±0.8^{b}	0.050	0.343	4.0 ±0.9^{b}	0.351	0.676	3.9 ±0.8^{b}	0.116	0.450	3.8 ±0.7^{*b}	0.013	0.217	< 0.001
5 min LM	2.8 ±0.4^{a}		0.213	3.1 ±0.7^{ac}		0.188	4.0 ±1.0^{b}		0.189	3.4 ±0.9^{abc}		0.843	3.7 ±0.9^{bc}		0.709	3.5 ±0.8^{abc}		0.510	3.2 ±0.7^{*ac}		0.720	< 0.001
30 min HM	3.3 ±0.7**	0.002		3.8 ±0.8	0.146		3.9 ±0.6	0.145		3.6 ±0.6	0.364		3.9 ±0.9	0.234		3.7 ±0.7	0.111		3.5 ±0.6	0.333		0.103
30 min LM	2.6 ±0.5^{**a}			17.1 ±64.7^{b}			3.6 ±0.7^{b}			3.4 ±0.9^{b}			3.6 ±0.8^{b}			3.3 ±0.9^{b}			3.3 ±0.9^{b}			0.012
**Motile BCF (Hz)**																						
5 min HM	22.3 ±2.9$^{** ** a}$	0.005	0.024	21.5 ±3.5^{ac}	0.082	0.343	17.8 ±2.6^{b}	0.848	0.616	19.3 ±2.7^{bc}	0.668	0.877	20.2 ±3.0^{*ac}	0.032	0.234	19.5 ±2.5^{*bc}	0.023	0.517	20.2 ±3.0^{abc}	0.775	0.935	< 0.001
5 min LM	19.2 ±2.9*		0.082	19.8 ±2.8		0.759	17.6 ±2.2		0.481	18.8 ±3.9		0.314	18.5 ±2.5*		0.572	17.8 ±2.4*		0.751	19.6 ±3.4		0.751	0.119
30 min HM	20.0 ±2.6$^{* ** ab}$	0.012		20.5 ±3.2^{a}	0.446		17.4 ±2.0^{b}	0.666		19.4 ±3.2^{ab}	0.101		19.2 ±3.1^{ab}	0.205		19.0 ±2.7^{ab}	0.131		20.2 ±3.2^{a}	0.741		0.043
30 min LM	17.7 ±2.9*			19.1 ±3.7			17.0 ±3.0			17.7 ±3.6			18.0 ±3.0			17.5 ±3.8			19.8 ±3.6			0.127

Supplementary Table S1. Continued…

**NP VCL (μm/s)**																							
5 min	**HM**	61.5 ±4.0	0.501	0.819	61.9 ±7.7	0.370	0.417	64.5 ±4.9	0.695	0.541	62.4 ±6.2	0.345	0.742	62.5 ±6.9	0.918	0.802	64.3 ±8.6	0.100	0.814	63.1 ±5.4	0.345	0.650	0.728
	**LM**	61.0 ±5.5		0.329	60.1 ±5.4		0.333	63.4 ±10.1		0.393	60.3 ±8.1		0.586	62.7 ±6.7		0.433	60.4 ±7.2		0.769	62.1 ±6.9		0.397	0.677
30 min	**HM**	61.0 ±7.4	0.267		**63.8 ±8.0***	**0.011**		63.2 ±6.8	0.316		61.7 ±8.5	0.911		62.0 ±7.3	0.656		61.4 ±13.7	0.181		61.1 ±8.1	0.552		0.939
	**LM**	57.7 ±8.7			**58.7 ±4.3***			61.0 ±5.9			61.4 ±5.2			61.0 ±8.4			61.1 ±7.2			64.2 ±9.5			0.287
**NP VAP (μm/s)**																							
5 min	**HM**	33.3 ±3.6	0.615	0.144	33.6 ±7.6	0.750	0.982	32.0 ±3.1	0.658	0.942	31.2 ±4.4	0.964	0.382	33.6 ±4.5	0.813	0.524	34.6 ±6.0	0.270	0.223	32.5 ±5.3	0.75	0.82	0.455
	**LM**	32.5 ±4.8		0.944	32.9 ±5.9		0.334	32.8 ±7.1		0.85	31.3 ±6.4		0.700	33.3 ±3.8		0.856	32.1 ±4.6		0.223	31.8 ±4.7		0.599	0.893
30 min	**HM**	30.8 ±5.5	0.430		33.6 ±6.7	0.202		31.9 ±4.2	0.801		32.7 ±6.6	0.691		32.9 ±6.9	0.401		32.4 ±5.5	0.623		32.3 ±7.4	0.812		0.9
	**LM**	32.3 ±5.3			31.5 ±3.7			31.5 ±4.8			32.0 ±5.8			32.9 ±8.1			33.2 ±5.0			32.8 ±7.5			0.951
**NP VSL (μm/s)**																							
5 min	**HM**	**22.7 ±5.9****	0.090	0.031	23.4 ±8.1	0.741	0.340	18.0 ±2.8	0.640	0.744	20.0 ±4.8	0.857	0.653	21.7 ±5.9	0.841	0.238	22.4 ±7.2	0.159	0.224	20.6 ±6.7	0.930	0.634	0.118
	**LM**	19.2 ±5.4		0.975	22.7 ±7.2		0.134	18.7 ±5.1		0.466	19.7 ±5.9		0.236	21.3 ±5.8		0.547	19.8 ±4.9		0.418	20.3 ±6.3		0.619	0.344
30 min	**HM**	**18.4 ±4.9****	0.704		21.4 ±6.0	0.364		17.7 ±2.9	0.854		19.8 ±6.3	0.401		19.7 ±5.0	0.859		20.2 ±4.3	0.597		20.7 ±6.2	0.733		0.383
	**LM**	19.1 ±5.8			19.9 ±5.0			17.4 ±4.7			17.6 ±6.0			20.1 ±7.7			20.9 ±4.6			21.4 ±7.4			0.251
**NP STR (%)**																							
5 min	**HM**	63.4 ±12.0	0.080	0.060	63.5 ±11.8	0.668	0.223	53.7 ±5.9	0.657	0.378	57.9 ±11.7	0.551	0.870	61.4 ±11.7	0.948	0.192	61.7 ±12.3	0.361	0.214	57.9 ±11.4	0.695	0.785	0.087
	**LM**	55.5 ±12.4		0.837	63.7 ±10.7		0.440	54.8 ±7.9		0.287	60.2 ±12.8		0.106	61.2 ±13.1		0.426	58.6 ±10.1		0.606	59.3 ±11.7		0.901	0.209
30 min	**HM**	56.1 ±9.1	0.938		59.5 ±10.1	0.888		51.9 ±5.8	0.893		57.4 ±10.5	0.307		56.7 ±12.3	0.684		57.9 ±6.6	0.375		58.7 ±8.4	0.740		0.288
	**LM**	56.4 ±11.2			58.2 ±11.9			51.5 ±9.7			52.7 ±15.0			58.2 ±11.7			60.1 ±8.9			59.7 ±9.8			0.117
**NP LIN (%)**																							
5 min	**HM**	**37.8 ±10.7**$^{** a}$	0.127	0.021	38.4 ±12.8^{a}	0.980	0.132	27.7 ±4.3^{b}	0.363	0.692	31.6 ±9.3^{ab}	0.631	0.832	36.6 ±11.3^{a}	0.485	0.142	35.6 ±11.0^{a}	0.413	0.236	32.7 ±10.8^{ab}	0.652	0.364	**0.023**
	**LM**	32.3 ±8.9		0.835	38.3 ±12.1		0.175	29.3 ±5.8		0.615	33.0 ±9.7		0.219	34.3 ±10.8		0.609	33.2 ±7.6		0.442	33.1 ±10.2		0.993	0.163
30 min	**HM**	**30.0 ±7.3****	0.268		33.4 ±8.2	0.874		27.1 ±4.3	0.943		32.2 ±8.5	0.316		32.0 ±9.3	0.809		31.6 ±6.2	0.146		33.4 ±9.3	0.905		0.224
	**LM**	33.5 ±10.2			33.9 ±9.2			28.0 ±8.5			29.4 ±9.8			32.7 ±9.8			35.0 ±7.1			33.1 ±9.9			0.201
**NP WOB (%)**																							
5 min	**HM**	53.5 ±7.2	0.803	0.182	54.8 ±10.8	0.863	0.429	49.5 ±3.7	0.264	0.868	50.6 ±6.2	0.667	0.255	54.1 ±7.9	0.691	0.626	54.4 ±8.0	0.564	0.107	51.0 ±7.2	0.819	0.622	0.166
	**LM**	54.1 ±6.9		0.267	55.3 ±9.1		0.454	51.4 ±5.9		0.948	51.5 ±7.4		0.700	53.2 ±7.3		0.810	53.2 ±5.1		0.585	51.5 ±8.1		0.997	0.516
30 min	**HM**	**50.4 ±5.5***	**0.007**		52.6 ±6.6	0.888		49.7 ±4.3	0.478		53.0 ±7.3	0.818		53.0 ±7.3	0.742		50.8 ±6.4	0.085		52.2 ±8.5	0.811		0.621
	**LM**	**57.0 ±7.2***			52.2 ±7.7			51.2 ±7.4			52.5 ±8.8			53.7 ±7.9			54.2 ±6.3			51.5 ±9.2			0.376
**MP VCL (μm/s)**																							
5 min	**HM**	124.7 ±24.8^{a}	0.926	0.285	138.8 ±23.9^{ab}	0.138	0.196	156.7 ±22.6^{b}	0.307	0.359	149.8 ±23.0^{b}	0.111	0.103	155.2 ±31.7^{b}	0.072	0.507	151.1 ±26.5^{b}	0.154	0.290	**148.8 ±20.8**$^{** b}$	**0.004**	0.304	**0.003**
	**LM**	123.9 ±18.0		0.527	127.8 ±25.5		0.118	148.7 ±22.7		0.121	138.0 ±24.6		0.564	139.4 ±26.2		0.383	140.5 ±22.0		0.33	**129.7 ±21.3****		0.240	**0.025**
30 min	**HM**	133.7 ±22.0	0.063		149.0 ±28.2	0.186		146.5 ±39.0	0.070		137.8 ±24.0	0.542		149.3 ±28.2	0.825		143.1 ±23.2	0.197		142.1 ±22.3	0.593		0.499
	**LM**	119.8 ±17.9			138.9 ±21.0			139.3 ±25.3			142.5 ±27.1			147.3 ±33.2			133.3 ±26.4			138.1 ±25.3			0.077
**MP VAP (μm/s)**																							
5 min	**HM**	**64.7 ±10.1***	**0.036**	0.836	**67.6 ±9.4***	**0.034**	0.581	67.8 ±7.5	0.174	0.554	64.1 ±11.9	0.335	0.466	**70.5 ±12.5****	**0.003**	0.629	**69.5 ±9.7****	**0.006**	0.229	**70.8 ±7.7*****	**<0.001**	0.229	0.207
	**LM**	**57.9 ±7.4***		0.520	**62.0 ±8.0***		0.279	63.4 ±5.3		0.704	62.8 ±6.8		0.091	**60.5 ±8.2****		0.192	**62.2 ±7.0****		0.561	**61.7 ±8.7*****		0.413	0.439
30 min	**HM**	**65.4 ±7.3****	**0.003**		69.2 ±10.0	0.061		65.2 ±16.4	0.058		66.7 ±9.1	0.917		68.9 ±9.2	0.161		65.7 ±10.8	0.105		67.7 ±9.2	0.159		0.798
	**LM**	**55.6 ±9.6**$^{** a}$			64.3 ±6.4^{ab}			63.3 ±5.8^{b}			67.0 ±9.4^{b}			64.5 ±11.5^{b}			60.7 ±9.0^{b}			63.8 ±8.3^{b}			**0.009**

Supplementary Table S1. Continued…

**MP VSL (μm/s)**																								
5 min	**HM**	**45.1 ±9.2***	**0.045**	0.575	46.4 ±9.2	0.253	0.590	40.7 ±5.9	0.089	0.875	**42.3 ±6.2***	0.882	0.045	**47.2 ±8.3****	**0.003**	0.350	**46.0 ±7.4****	**0.007**	0.107	46.1 ±6.1	0.063	0.156	**0.009**
	**LM**	**39.0 ±6.8***		0.884	43.6 ±7.1		0.849	37.1 ±6.1		0.142	42.0 ±6.9		0.430	**39.1 ±9.3****		0.216	**40.0 ±6.7****		0.366	40.7 ±10.8		0.542	0.204
30 min	**HM**	**43.6 ±5.0****	**0.002**		45.0 ±7.3	0.373		40.4 ±4.9	0.921		**46.0 ±5.8***	0.209		45.0 ±6.9	0.259		42.5 ±6.6	0.868		44.0 ±5.8	0.441		0.116
	**LM**	**37.8 ±6.4****			43.2 ±6.4			40.2 ±6.2			43.6 ±6.8			42.4 ±8.3			42.1 ±8.7			42.4 ±7.3			0.252
**MP STR (%)**																								
5 min	**HM**	70.3 ±12.8^{a}	0.542	0.490	68.8 ±10.8^{a}	0.739	0.293	59.4 ±8.3^{b}	0.796	0.517	**63.9 ±7.4$^{*\ ab}$**	0.287	0.025	67.7 ±9.2^{ab}	0.364	0.362	66.1 ±10.3^{ab}	0.645	0.411	66.0 ±7.5^{ab}	0.191	0.686	**0.024**
	**LM**	67.7 ±9.8		0.944	69.9 ±11.0		0.595	59.6 ±9.3		0.270	66.9 ±10.7		0.724	64.8 ±12.2		0.665	64.8 ±8.9		0.263	66.6 ±16.2		0.330	0.193
30 min	**HM**	67.5 ±9.1	0.993		65.7 ±9.0	0.294		60.1 ±6.4	0.292		**69.1 ±7.2***	0.194		65.7 ±9.6	0.307		62.5 ±14.0	0.074		65.1 ±7.2	0.525		0.084
	**LM**	67.5 ±7.8			68.4 ±7.8			63.9 ±13.1			65.8 ±8.8			66.4 ±11.5			68.3 ±11.6			66.8 ±9.1			0.839
**MP LIN (%)**																								
5 min	**HM**	42.4 ±13.0^{a}	0.103	0.187	39.0 ±12.5^{ab}	0.761	0.112	28.6 ±7.7^{d}	0.679	0.195	**31.8 ±6.5$^{*\ dc}$**	0.243	0.033	34.6 ±9.3^{abc}	0.359	0.645	34.3 ±9.1^{bc}	0.542	0.833	35.0 ±8.0^{ac}	0.684	0.742	**0.003**
	**LM**	35.7 ±8.4^{ab}		0.890	40.1 ±11.7^{a}		0.291	27.7 ±7.0^{b}		0.391	34.7 ±9.2^{ab}		0.964	31.9 ±11.0^{ab}		0.414	32.7 ±9.5^{ab}		0.312	35.8 ±12.5^{ab}		0.783	**0.008**
30 min	**HM**	37.4 ±8.7	0.968		34.2 ±10.0	0.054		29.3 ±4.4	0.756		**37.4 ±7.8***	0.146		34.2 ±10.6	0.700		33.6 ±5.0	0.390		34.7 ±6.7	0.952		0.068
	**LM**	35.4 ±6.9			36.9 ±7.9			31.7 ±10.5			34.5 ±8.2			33.3 ±9.9			35.7 ±10.0			34.9 ±8.7			0.068
**MP WOB (%)**																								
5 min	**HM**	**56.1 ±8.9$^{*\ a}$**	**0.048**	0.159	52.9 ±9.8^{ac}	0.956	0.210	45.4 ±5.5^{b}	0.770	0.183	**46.8 ±4.9$^{**\ bd}$**	0.243	0.015	48.5 ±6.6^{bcd}	0.157	0.904	49.4 ±6.2^{dc}	0.258	0.945	50.3 ±6.4^{ad}	0.904	0.555	**0.003**
	**LM**	**50.0 ±7.5$^{*\ ab}$**		0.862	53.0 ±8.9^{a}		0.267	42.7 ±11.1^{b}		0.187	48.8 ±6.4^{ab}		0.351	46.5 ±8.7^{ab}		0.382	47.3 ±5.6^{ab}		0.374	50.9 ±9.0^{a}		0.612	**0.005**
30 min	**HM**	52.3 ±5.4	0.214		49.6 ±7.3	0.691		47.7 ±3.9	0.776		**51.7 ±5.8****	0.699		49.4 ±7.6	0.388		49.5 ±4.8	0.859		50.7 ±5.4	0.576		0.304
	**LM**	49.5 ±6.9			50.4 ±6.3			47.1 ±6.5			50.5 ±5.7			47.3 ±6.9			49.1 ±7.7			49.6 ±6.6			0.522
**MP ALH (μm)**																								
5 min	**HM**	3.6 ±0.9^{a}	0.728	0.281	3.9 ±0.9^{ac}	0.322	0.114	4.8 ±0.9^{b}	0.602	0.723	4.5 ±0.8^{bc}	0.196	0.053	4.6 ±1.1^{bc}	0.285	0.664	4.5 ±1.0^{bc}	0.561	0.430	**4.3 ±0.8$^{*\ bc}$**	**0.025**	0.638	**0.002**
	**LM**	3.7 ±0.6^{a}		0.762	3.6 ±0.9^{a}		0.116	4.6 ±0.9^{b}		0.567	4.2 ±0.9^{ab}		0.609	4.3 ±1.0^{ab}		0.429	4.3 ±0.8^{ab}		0.289	**3.7 ±0.9$^{*\ a}$**		0.111	**0.002**
30 min	**HM**	3.9 ±0.8	0.253		4.4 ±0.9	0.241		4.7 ±0.6	0.404		4.0 ±0.8	0.274		4.5 ±1.0	0.857		4.2 ±0.8	0.387		4.2 ±0.7	0.924		0.110
	**LM**	3.6 ±0.7			4.0 ±0.8			4.4 ±1.1			4.3 ±0.9			4.5 ±1.1			4.0 ±0.9			4.2 ±0.9			0.084
**MP BCF (Hz)**																								
5 min	**HM**	**22.0 ±3.4$^{*\ **\ a}$**	**0.017**	0.009	20.3 ±3.7^{ac}	0.290	0.089	17.2 ±2.8^{b}	0.667	0.540	17.6 ±2.6^{b}	0.97	0.243	18.8 ±2.7^{bc}	0.234	0.127	18.3 ±2.7^{bc}	0.116	0.664	19.3 ±2.7^{bc}	0.487	0.132	**< 0.001**
	**LM**	**19.4 ±2.2$^{*\ ab}$**		0.312	19.1 ±3.7^{ab}		0.700	16.8 ±2.3^{a}		0.843	17.6 ±3.6^{ab}		0.586	18.0 ±2.2^{ab}		0.117	17.1 ±2.5^{a}		0.577	**20.0 ±4.0$^{*\ b}$**		0.044	**0.006**
30 min	**HM**	**19.2 ±2.3****	0.442		18.6 ±3.5	0.690		16.4 ±1.7	0.904		18.5 ±2.5	0.08		17.8 ±3.4	0.346		17.9 ±2.7	0.991		18.1 ±2.6	0.629		0.165
	**LM**	18.4 ±2.8			19.7 ±8.0			16.9 ±4.4			17.0 ±3.0			16.5 ±3.8			17.5 ±2.8			**17.6 ±3.4***			0.292
**RP VCL (μm/s)**																								
5 min	**HM**	137.2 ±18.2^{a}	0.501	0.951	145.9 ±16.1^{ab}	0.601	0.315	152.2 ±11.3^{ab}	0.104	0.301	148.2 ±15.6^{ab}	0.597	0.662	154.4 ±18.4^{b}	0.401	0.665	153.0 ±19.6^{b}	0.118	0.280	147.7 ±14.3^{ab}	0.514	0.801	**0.041**
	**LM**	133.6 ±13.5		0.432	143.3 ±17.4		0.468	144.9 ±14.1		0.683	142.8 ±18.7		0.947	149.5 ±20.2		0.961	142.6 ±23.7		0.865	144.9 ±15.2		0.794	0.33
30 min	**HM**	137.5 ±13.3	0.226		150.9 ±17.1	0.495		148.4 ±9.1	0.304		150.2 ±16.0	0.163		152.0 ±18.0	0.595		146.9 ±17.7	0.554		146.6 ±14.1	0.501		0.124
	**LM**	130.2 ±18.9			147.2 ±18.5			142.3 ±21.8			142.4 ±20.6			149.3 ±16.1			143.6 ±17.7			143.7 ±13.8			0.116
**RP VAP (μm/s)**																								
5 min	**HM**	79.6 ±11.7	0.105	0.837	79.8 ±12.3	0.214	0.264	71.2 ±17.7	0.692	0.183	77.2 ±10.9	0.530	0.296	**81.3 ±11.7****	**0.005**	0.954	**80.8 ±10.0***	**0.027**	0.701	**81.3 ±10.3***	**0.042**	0.492	0.139
	**LM**	73.5 ±11.1		0.861	75.4 ±11.2		0.419	72.7 ±7.6		0.964	75.2 ±9.8		0.779	**72.6 ±8.1****		0.047	**74.4 ±8.7***		0.275	**74.5 ±11.8***		0.181	0.947
30 min	**HM**	78.8 ±8.8	0.084		83.7 ±10.9	0.075		78.1 ±6.7	0.069		**80.7 ±10.5***	**0.035**		81.1 ±11.4	0.344		79.6 ±10.9	0.334		83.4 ±10.3	0.118		0.519
	**LM**	72.9 ±9.3			78.0 ±9.8			72.8 ±9.2			**74.4 ±8.8***			78.0 ±9.6			76.9 ±6.3			78.8 ±7.9			0.171

Supplementary Table S1. Continued…

**RP VSL (μm/s)**																							
5 min	HM	71.7 ±11.7	0.166	0.920	71.9 ±12.5	0.226	0.369	66.3 ±7.6	0.422	0.359	68.6 ±10.5	0.928	0.289	**72.3 ±11.1****	**0.004**	0.782	**71.9 ±8.8***	**0.039**	0.617	72.7 ±10.2	0.061	0.437	0.436
	LM	66.4 ±11.1		0.895	67.6 ±11.5		0.329	64.2 ±7.6		0.921	68.8 ±8.6		0.326	**63.8 ±7.4****		0.036	**66.7 ±7.4***		0.485	66.4 ±12.2		0.150	0.643
30 min	HM	71.3 ±9.1	0.108		**75.1 ±10.8***	**0.013**		68.7 ±7.0	0.154		**72.0 ±10.4***	**0.049**		73.2 ±11.8	0.242		70.5 ±10.0	0.371		75.2 ±10.5	0.166		0.383
	LM	65.1 ±11.1			**69.1 ±9.1***			64.5 ±9.4			**66.3 ±8.3***			69.4 ±9.5			68.1 ±6.3			71.1 ±7.5			0.334
**RP STR (%)**																							
5 min	HM	90.5 ±2.0^{a}	0.580	0.595	89.9 ±2.6^{a}	0.318	0.836	87.4 ±2.2^{b}	0.480	0.378	88.5 ±1.9^{ab}	0.199	0.570	88.7 ±2.1^{ab}	0.211	0.753	88.8 ±1.6^{ab}	0.868	0.386	89.1 ±2.4^{ab}	0.484	0.364	**<0.001**
	LM	89.9 ±3.1		0.458	89.2 ±2.6		0.717	86.5 ±7.8		0.974	89.6 ±3.2		0.427	87.8 ±2.5		0.364	88.9 ±3.4		0.609	88.6 ±3.0		0.089	0.112
30 min	HM	90.1 ±2.4	0.340		89.8 ±2.7	0.270		87.8 ±1.9	0.809		88.9 ±2.2	0.986		88.5 ±1.8	0.993		89.2 ±1.6	0.221		89.8 ±2.3	0.764		**0.024**
	LM	88.8 ±4.7			88.9 ±2.5			88.2 ±3.5			88.9 ±2.5			88.5 ±2.4			88.5 ±2.3			90.0 ±2.2			0.334
**RP LIN (%)**																							
5 min	HM	54.4 ±7.3^{a}	0.123	0.624	50.9 ±8^{ac}	0.214	0.985	44.9 ±5.9^{b}	0.734	0.160	47.4 ±5.1^{bc}	0.264	0.715	**48.3 ±6.4**$^{*\ bc}$	**0.029**	0.975	49.1 ±4.7^{ab}	0.889	0.936	50.4 ±6.1^{ab}	0.071	0.271	**<0.001**
	LM	50.5 ±8.0		0.965	48.0 ±8.0		0.743	45.6 ±5.9		0.704	49.9 ±8.7		0.299	**44.2 ±5.7***		0.060	48.8 ±9.3		0.936	**46.7 ±7.1***		0.043	0.112
30 min	HM	53.2 ±6.2^{a}	0.604		50.9 ±6.2^{ab}	0.270		47.3 ±3.9^{b}	0.138		48.1 ±6.1^{ab}	0.848		48.3 ±5.1^{ab}	0.776		49.0 ±3.7^{ab}	0.466		52.4 ±6.2^{ab}	0.369		**0.005**
	LM	51.5 ±11.6			48.7 ±6.8			46.5 ±8.2			47.8 ±4.5			47.8 ±6.6			48.7 ±5.6			**50.8 ±5.0***			0.112
**RP WOB (%)**																							
5 min	HM	59.6 ±7.1^{a}	0.123	0.659	56.2 ±7.5^{ac}	0.459	0.849	51.0 ±5.5^{b}	0.811	0.121	53.2 ±4.6^{bc}	0.271	0.265	**54.0 ±6.0**$^{*\ bc}$	**0.022**	0.869	54.3 ±4.1^{bc}	0.993	0.829	**56.1 ±5.5**$^{*\ ac}$	**0.040**	0.387	**0.011**
	LM	55.7 ±7.3		0.895	54.7 ±5.9		0.896	51.5 ±5.4		0.673	55.3 ±7.4		0.296	**50.0 ±5.4****		0.029	54.4 ±8.3		0.829	**52.3 ±6.5****		0.038	0.054
30 min	HM	58.6 ±5.5^{a}	0.661		56.5 ±5.5^{ac}	0.414		53.7 ±3.4^{b}	0.098		54.8 ±4.5^{bc}	0.303		54.3 ±4.7^{bc}	0.890		54.6 ±3.4^{bcd}	0.512		57.5 ±5.6^{ad}	0.367		**0.013**
	LM	57.3 ±10.0			54.9 ±7.3			52.4 ±7.1			53.4 ±4.0			**54.0 ±6.4***			54.7 ±5.3			**56.1 ±4.3***			0.127
**RP ALH (μm)**																							
5 min	HM	3.3 ±0.6^{a}	0.843	0.254	3.8 ±0.8^{b}	0.968	0.506	4.3 ±0.7^{b}	0.274	0.658	4.1 ±0.7^{b}	0.244	0.853	4.1 ±0.8^{b}	0.594	0.735	4.1 ±0.8^{b}	0.568	0.735	3.9 ±0.6^{b}	0.666	0.988	**<0.001**
	LM	3.4 ±0.7		0.570	3.8 ±0.7		0.227	4.0 ±0.8		0.497	3.8 ±0.9		0.593	4.3 ±1.0		0.902	4.0 ±1.0		0.913	4.0 ±0.7		0.666	0.154
30 min	HM	3.5 ±0.5^{a}	0.841		4.0 ±0.7^{ab}	0.703		4.2 ±0.5^{b}	0.942		4.1 ±0.6^{b}	0.298		4.2 ±0.7^{b}	0.870		4.1 ±0.6^{b}	0.794		3.9 ±0.7^{ab}	0.967		**0.017**
	LM	3.4 ±1.0			4.0 ±0.6			4.2 ±0.7			3.9 ±0.7			4.2 ±0.7			4.0 ±0.8			3.9 ±0.7			0.059
**RP BCF (Hz)**																							
5 min	HM	**25.7 ±2.6**$^{*\ a}$	0.105	0.034	24.1 ±3.0^{ac}	0.189	0.191	20.5 ±3.4^{b}	0.342	0.925	22.1 ±2.9^{bc}	0.461	0.761	22.6 ±3.0^{bc}	0.256	0.163	22.1 ±2.7^{bc}	0.774	0.332	22.6 ±2.8^{bc}	0.138	0.871	**<0.001**
	LM	24.0 ±2.7		0.662	22.8 ±3.3		0.666	21.6 ±3.5		0.061	22.8 ±3.7		0.209	21.5 ±3.0		0.432	21.8 ±5.0		0.844	20.0 ±6.1		0.098	0.097
30 min	HM	**23.7 ±2.7***	0.778		22.8 ±3.4	0.622		20.4 ±2.8	0.407		21.8 ±3.5	0.700		20.7 ±4.9	0.733		21.4 ±2.2	0.823		22.8 ±3.3	0.723		0.097
	LM	23.3 ±5.3			21.3 ±5.7			19.6 ±2.7			20.7 ±5.0			20.8 ±3.4			21.6 ±2.0			23.1 ±3.4			0.110

ALH, average path velocity; BCF, beat cross frequency; CAP, capacitating-HTF; HD-C, HD capacitation medium; HTF, human tubal fluid; HM, high motile subpopulation; LIN, linearity; LM, low motile subpopulation; MP, medium-progressive; min, minutes; MYO, myo-inositol; NP, non-progressive; PRG, progesterone; Prog, progressive; RP, rapid-progressive; SD, standard deviation; SP *p*, t-test between subpopulations; STR, straightness; TP *p*, t test between time points; VAP, average path velocity; VCL, curvilinear velocity; VSL, straight-line velocity; WOB, wobble. Values labelled in bold with an asterisks in the same column were significantly different between the HM and LM subpopulations for individual media and SCA parameters **(*p <0.05, **p <0.01 and ***p <0.001)**. Values labelled in bold and red with an asterisks in the same column were significantly different between the 5 and 30 minutes for individual subpopulations and media **(*p <0.05 and **p <0.01).** Student's t-test was used or the Mann-Whitney test when normal distribution was absent. [a, b,c,d] Values labelled with different superscript letters in the same row were significantly different between the various media for individual subpopulations and SCA parameters. One-way ANOVA was used for parametric distributions or Kruskal-Wallis test for non-parametric distributions.

Supplementary Table S2. Comparison of mean SCA motility and kinematic parameters at 5 and 30 minutes for both the low motile (LM) and high motile (HM) sperm subpopulations after treatment with HTF, CAP, 20 nM, 100 nM and 1 μM dopamine (mean ± SD) (n = 20).

			HTF			CAP			20 nM DOPA			100 nM DOPA			1 μM DOPA			
			Mean ± SD	SP *p*	TP *p*	Mean ± SD	SP *p*	TP *p*	Mean ± SD	SP *p*	TP *p*	Mean ± SD	SP *p*	TP *p*	Mean ± SD	SP *p*	TP *p*	ANOVA
**Prog (%)**																		
5 min	HM		**44.5 ± 23.5*****	**<0.001**	0.251	**51.4 ± 21.1*****	**<0.001**	0.944	**57.4 ± 27.1*****	**<0.001**	0.415	**54.5 ± 22.2*****	**<0.001**	0.318	57.0 ± 25.0	0.174	0.204	0.306
	LM		**18.4 ± 15.7*****		0.497	**26.7 ± 17.3*****		0.190	**29.2 ± 17.5*****		0.849	**28.7 ± 18.2***		0.550	31.1 ± 25.5		0.968	0.140
30 min	HM		**37.0 ± 22.5*****	**<0.001**		**51.0 ± 25.4*****	**<0.001**		**51.4 ± 25.6****	**0.003**		**48.1 ± 24.7*****	**0.011**		47.4 ± 27.6	0.968		0.257
	LM		**14.9 ± 14.3^{***a}**			**21.7 ± 20.0^{***ab}**			**30.3 ± 21.0^{**b}**			**30.7 ± 20.0^{*ab}**			30.5 ± 22.0^{b}			**0.017**
**Motile (%)**																		
5 min	HM		**60.8 ± 24.0*****	**<0.001**	0.170	**67.4 ± 19.6*****	**<0.001**	0.854	**66.0 ± 26.2*****	**<0.001**	0.388	**65.6 ± 22.5*****	**<0.001**	0.377	66.2 ± 22.3	0.273	0.222	0.875
	LM		**31.8 ± 24.5*****		0.646	**40.9 ± 21.9*****		0.306	**39.6 ± 21.2*****		0.667	**40.3 ± 22.3*****		0.781	41.4 ± 26.4		0.857	0.583
30 min	HM		**51.3 ± 24.4****	**0.001**		**64.0 ± 26.6*****	**<0.001**		**60.5 ± 27.2***	**0.011**		**59.0 ± 25.8***	**0.015**		57.9 ± 24.9	0.857		0.521
	LM		**28.5 ± 20.5****			**34.0 ± 25.0*****			**42.4 ± 23.6***			**42.2 ± 22.6***			40.5 ± 23.9			0.167
**RP (%)**																		
5 min	HM		**18.9 ± 14.4*****	**<0.001**	0.443	**25.2 ± 17.8*****	**<0.001**	0.778	**27.5 ± 17.7****	**0.003**	0.907	**28.4 ± 15.1****	**0.001**	0.497	30.5 ± 20.3	0.946	0.946	0.160
	LM		**6.6 ± 7.3*****		0.856	**10.7 ± 8.9*****		0.248	**12.8 ± 8.6****		0.771	**13.1 ± 11.7****		0.606	16.2 ± 17.5		0.834	0.056
30 min	HM		**15.0 ± 11.2^{**a}**	**0.006**		**26.5 ± 17.8^{***b}**	**<0.001**		**28.7 ± 17.9^{***b}**	**<0.001**		**25.4 ± 16.6^{**b}**	**0.006**		30.2 ± 19.9^{b}	0.834		**0.017**
	LM		**6.3 ± 7.5****			**9.4 ± 11.9*****			**13.4 ± 11.6*****			**13.9 ± 9.8****			14.4 ± 13.9			0.056
**MP (%)**																		
5 min	HM		**25.7 ± 18.7****	**0.003**	0.727	**26.3 ± 17.7***	**0.024**	0.977	**29.9 ± 15.9****	**0.002**	0.106	**26.6 ± 14.7****	**0.002**	0.516	**26.5 ± 15.6****	**0.004**	0.060	0.908
	LM		**11.8 ± 9.4****		0.194	**15.9 ± 11.0***		0.236	**16.5 ± 10.3****		0.915	**15.6 ± 7.9****		0.910	**14.9 ± 11.3****		0.704	0.501
30 min	HM		**22.0 ± 13.6*****	**<0.001**		**24.5 ± 15.1****	**0.002**		22.7 ± 14.8	0.127		22.7 ± 12.5	0.114		19.7 ± 15.3	0.704		0.836
	LM		**8.6 ± 7.8^{***a}**			**12.3 ± 10.2^{**ab}**			16.9 ± 11.5^{ab}			16.8 ± 12.2^{ab}			16.1 ± 11.9^{b}			**0.035**
**NP (%)**																		
5 min	HM		16.3 ± 8.3^{a}	0.101	0.574	16.0 ± 12.2^{ac}	0.946	0.388	8.6 ± 4.5^{b}	0.287	0.692	11.1 ± 5.4^{bc}	0.758	0.503	9.2 ± 7.3^{c}	0.290	0.290	**< 0.001**
	LM		13.4 ± 12.5		0.976	14.2 ± 7.9		0.286	10.4 ± 7.2		0.421	11.7 ± 8.1		0.828	10.2 ± 4.5		0.899	0.441
30 min	HM		14.3 ± 8.4	0.522		13.0 ± 9.6	0.854		9.1 ± 4.8	0.100		10.9 ± 7.5	0.665		11.5 ± 8.8	0.653		0.305
	LM		13.7 ± 10.8			12.3 ± 9.0			12.1 ± 7.6			11.5 ± 6.4			10.0 ± 6.0			0.920
**Rapid (%)**																		
5 min	HM		**29.9 ± 19.8^{***a}**	**<0.001**	0.873	**40.9 ± 22.3^{***ab}**	**<0.001**	0.557	**50.0 ± 27.0^{***b}**	**<0.001**	0.520	**47.1 ± 21.0^{***b}**	**<0.001**	0.491	49.3 ± 24.2^{b}	0.240	0.301	**0.012**
	LM		**11.6 ± 10.3^{***a}**		0.711	**19.6 ± 14.2^{***b}**		0.313	**22.2 ± 13.4^{***b}**		0.933	**22.8 ± 16.0^{***b}**		0.657	27.9 ± 23.6^{b}		0.818	**0.027**
30 min	HM		**30.8 ± 20.8*****	**<0.001**		**45.0 ± 26.1*****	**<0.001**		**45.3 ± 24.7*****	**<0.001**		**42.9 ± 23.6****	**0.007**		41.7 ± 26.8	0.818		0.211
	LM		**11.3 ± 11.9^{***a}**			**16.8 ± 16.0^{***ac}**			**22.5 ± 15.4^{***bc}**			**24.8 ± 17.1^{**bc}**			29.0 ± 23.9^{b}			**0.005**
**Medium (%)**																		
5 min	HM		**18.1 ± 14.3$^{**\ **\ a}$**	**0.007**	**0.009**	13.4 ± 9.6^{ab}	0.273	0.148	9.4 ± 7.9^{b}	0.961	0.786	10.2 ± 5.2^{ab}	0.107	0.207	9.4 ± 8.0^{b}	0.295	0.295	**0.046**
	LM		**9.2 ± 7.7****		0.197	10.7 ± 7.2		0.133	9.4 ± 7.4		0.522	8.0 ± 6.5		0.543	5.5 ± 5.0		0.180	0.086
30 min	HM		**9.6 ± 7.9****	0.165		9.3 ± 7.9	0.560		9.1 ± 8.0	0.265		8.7 ± 6.5	0.812		7.5 ± 7.0	0.180		0.876
	LM		6.7 ± 5.6			7.7 ± 6.8			10.8 ± 7.7			8.6 ± 5.7			6.9 ± 4.6			0.130
**Slow (%)**																		
5 min	HM		12.9 ± 7.8^{a}	0.148	0.327	13.1 ± 12.0^{a}	0.839	0.304	6.5 ± 3.9^{b}	0.289	0.719	7.1 ± 4.9^{b}	0.273	0.684	7.5 ± 6.0^{b}	0.594	0.594	**0.001**
	LM		11.0 ± 11.0		0.968	10.6 ± 6.2		0.256	8.0 ± 5.8		0.528	9.5 ± 7.3		0.796	8.0 ± 4.2		0.779	0.533
30 min	HM		10.9 ± 6.9	0.689		9.7 ± 7.0	0.823		**6.2 ± 3.2***	**0.036**		7.4 ± 4.5	0.451		8.4 ± 6.1	0.654		0.194
	LM		10.6 ± 8.7			9.5 ± 7.9			**9.0 ± 5.7***			8.6 ± 5.5			7.7 ± 5.0			0.656

Supplementary Table S2. Continued…

**Motile VCL (µm/s)**																	
5 min	HM	101.0 ± 21.0$^{*\,a}$	0.015	0.144	115.5 ± 26.2$^{*\,b}$	0.026	0.083	137.2 ± 29.0$^{***\,c}$	<0.001	0.393	134.1 ± 22.7$^{**\,c}$	0.005	0.299	132.6 ± 25.4$^{*\,c}$	0.027	0.865	<0.001
	LM	87.6 ± 16.3$^{*\,a}$		0.554	100.9 ± 18.1$^{*\,b}$		0.909	107.3 ± 18.0$^{***\,bc}$		0.872	115.4 ± 21.8$^{**\,c}$		0.599	114.9 ± 29.1$^{*\,c}$		0.862	<0.001
30 min	HM	110.7 ± 24.9$^{***\,a}$	<0.001		128.9 ± 27.3$^{***\,b}$	<0.001		130.8 ± 23.5$^{***\,b}$	<0.001		129.4 ± 27.2$^{**\,b}$	0.009		131.2 ± 31.2^{ab}	0.089		0.038
	LM	84.5 ± 19.3$^{***\,a}$			100.2 ± 18.4$^{***\,b}$			108.1 ± 17.6$^{***\,bc}$			111.7 ± 17.4$^{**\,bc}$			116.3 ± 28.6^{c}			<0.001
**Motile VAP (µm/s)**																	
5 min	HM	56.4 ± 9.6$^{***\,a}$	<0.001	0.320	59.1 ± 11.6$^{*\,ab}$	0.012	0.565	63.7 ± 10.1$^{***\,ab}$	<0.001	0.836	63.5 ± 9.7$^{**\,b}$	0.002	0.272	64.9 ± 11.3^{b}	0.467	0.392	0.022
	LM	45.9 ± 10.5$^{***\,a}$		0.147	51.6 ± 8.5$^{*\,b}$		0.244	53.7 ± 5.6$^{***\,bc}$		0.85	55.8 ± 6.9$^{**\,c}$		0.267	55.5 ± 14.1^{bc}		0.897	<0.001
30 min	HM	53.4 ± 10.9$^{***\,a}$	<0.001		60.9 ± 10.4$^{***\,b}$	<0.001		63.1 ± 9.0$^{***\,b}$	<0.001		60.6 ± 8.7$^{**\,b}$	0.004		61.8 ± 13.9^{b}	0.064		0.018
	LM	41.8 ± 9.1$^{***\,a}$			48.6 ± 9.0$^{***\,b}$			53.3 ± 7.7$^{***\,b}$			53.5 ± 7.5$^{**\,b}$			54.9 ± 11.6^{b}			<0.001
**Motile VSL (µm/s)**																	
5 min	HM	46.0 ± 10.7$^{**\,*}$	0.001	0.014	47.4 ± 13.6*	0.023	0.734	49.2 ± 11.0*	0.018	0.541	48.9 ± 10.2	0.076	0.677	51.3 ± 12.7*	0.013	0.403	0.582
	LM	34.7 ± 12.7$^{**\,a}$		0.121	38.4 ± 10.8$^{*\,ab}$		0.442	41.7 ± 7.1$^{*\,b}$		0.963	42.6 ± 8.3^{b}		0.197	41.5 ± 14.0$^{*\,ab}$		0.916	0.039
30 min	HM	38.4 ± 10.5$^{*\,*\,a}$	0.005		47.3 ± 11.1$^{***\,b}$	<0.001		50.3 ± 9.4$^{**\,b}$	0.002		47.1 ± 7.5$^{**\,b}$	0.004		48.0 ± 14.6^{b}	0.071		0.002
	LM	29.5 ± 10.5$^{*\,a}$			36.1 ± 9.8$^{***\,b}$			41.6 ± 9.8$^{**\,b}$			39.9 ± 9.2$^{**\,b}$			41.2 ± 110^{b}			<0.001
**Motile STR (%)**																	
5 min	HM	57.8 ± 8.5$^{*\,***}$	0.036	<0.001	53.7 ± 7.7	0.140	0.436	49.5 ± 8.2	0.992	0.282	49.6 ± 6.2	0.959	0.612	51.9 ± 7.7*	0.034	0.327	0.410
	LM	54.0 ± 8.4*		0.145	52.9 ± 7.0		0.368	52.6 ± 6.6		0.793	51.8 ± 6.2		0.375	50.8 ± 7.3*		0.734	0.623
30 min	HM	49.9 ± 4.9$^{***\,a}$	0.298		49.2 ± 6.6$^{*\,ab}$	0.047		50.6 ± 5.5^{b}	0.255		49.0 ± 4.8^{ab}	0.124		48.7 ± 5.9^{ab}	0.465		0.019
	LM	50.9 ± 8.1			50.0 ± 6.0*			51.0 ± 6.6			50.2 ± 5.4			49.6 ± 5.5			0.060
**Motile LIN (%)**																	
5 min	HM	78.7 ± 10.7$^{*\,***\,a}$	0.042	<0.001	76.8 ± 12.3^{ab}	0.284	0.097	74.2 ± 9.4^{b}	0.229	0.367	73.8 ± 8.1^{b}	0.442	0.818	76.1 ± 9.0^{ab}	0.233	0.115	0.020
	LM	70.7 ± 15.1*		0.080	70.9 ± 12.0		0.230	74.2 ± 8.8		0.568	73.7 ± 10.3		0.235	70.1 ± 10.5		0.952	0.828
30 min	HM	68.3 ± 9.6***	0.650		74.3 ± 9.8	0.489		76.7 ± 7.3	0.809		74.8 ± 4.8	0.716		72.5 ± 12.1	0.993		0.317
	LM	64.8 ± 12.8			68.8 ± 9.1			73.7 ± 10.9			70.9 ± 11.4			71.1 ± 8.9			0.365
**Motile WOB (%)**																	
5 min	HM	47.6 ± 12.2$^{***\,a}$	0.115	<0.001	43.1 ± 11.5$^{*\,ab}$	0.695	0.032	39.1 ± 11.4^{b}	0.079	0.299	38.3 ± 7.8^{b}	0.205	0.703	41.2 ± 10.1^{b}	0.630	0.218	0.001
	LM	40.2 ± 13.0		0.201	39.7 ± 10.5		0.128	41.0 ± 8.9		0.389	40.1 ± 8.5		0.328	37.6 ± 10.1		0.504	0.613
30 min	HM	35.5 ± 7.3***	0.606		38.1 ± 9.0*	0.687		40.1 ± 7.6	0.819		37.8 ± 5.2	0.422		36.8 ± 9.1	0.478		0.765
	LM	34.4 ± 9.1			36.5 ± 7.9			39.5 ± 9.7			37.1 ± 8.5			36.8 ± 7.3			0.929
**Motile ALH (µm)**																	
5 min	HM	2.6 ± 0.8$^{**\,a}$	0.655	0.003	3.0 ± 0.8$^{*\,a}$	0.367	0.006	3.8 ± 1.0$^{**\,b}$	0.001	0.780	3.7 ± 0.6$^{*\,b}$	0.030	0.727	3.6 ± 0.8^{b}	0.092	0.550	<0.001
	LM	2.4 ± 0.5^{a}		0.313	2.8 ± 0.6^{b}		0.447	3.0 ± 0.6$^{**\,b}$		0.622	3.2 ± 0.8$^{*\,b}$		0.734	3.2 ± 0.8^{b}		0.551	<0.001
30 min	HM	3.2 ± 0.7$^{***\,**}$	<0.001		3.6 ± 0.9$^{***\,*}$	<0.001		3.7 ± 0.7**	0.001		3.7 ± 0.7*	0.020		3.7 ± 0.9	0.105		0.148
	LM	2.5 ± 0.5$^{***\,a}$			2.9 ± 0.5$^{***\,b}$			3.0 ± 0.6$^{**\,b}$			3.3 ± 0.6$^{*\,b}$			3.3 ± 0.8^{b}			<0.001
**Motile BCF (Hz)**																	
5 min	HM	23.2 ± 4.4***	0.206	<0.001	23.2 ± 4.5*	0.060	0.015	21.3 ± 4.3	0.728	0.230	20.7 ± 3.8	0.951	0.268	21.9 ± 3.6*	0.121	0.024	0.128
	LM	21.6 ± 4.3**		0.004	20.9 ± 3.7		0.233	20.9 ± 2.9		0.298	20.7 ± 3.2		0.071	20.3 ± 3.5		0.502	0.758
30 min	HM	18.9 ± 2.8***	0.382		20.4 ± 3.0*	0.464		20.0 ± 2.8	0.981		19.7 ± 2.7	0.392		19.7 ± 2.8*	0.927		0.398
	LM	18.0 ± 4.0**			19.8 ± 3.0			20.0 ± 2.8			19.0 ± 3.1			19.6 ± 2.9			0.179

Supplementary Table S2. Continued…

NP VCL (μm/s)																		
5 min	HM	60.9 ± 7.7**	0.007	0.250	59.4 ± 10.4	0.372	0.706	60.0 ± 9.9	0.108	0.757	64.6 ± 13.5	0.138	0.447	59.1 ± 10.0	0.832	0.911	0.350
	LM	55.3 ± 10.1**		0.111	58.5 ± 11.9		0.254	56.0 ± 7.5**		0.006	59.6 ± 9.4		0.415	59.5 ± 10.4		0.358	0.433
30 min	HM	58.4 ± 7.4	0.631		58.3 ± 10.9	0.289		61.6 ± 7.7	0.472		62.2 ± 7.7	0.685		58.8 ± 8.1	0.276		0.311
	LM	57.6 ± 8.0			61.4 ± 9.5			62.1 ± 7.4**			61.4 ± 4.9			61.2 ± 6.6			0.244
NP VAP (μm/s)																		
5 min	HM	33.9 ± 6.1**	0.001	0.076	32.9 ± 7.4**	0.190	0.008	30.2 ± 7.1	0.885	0.389	29.7 ± 6.0	0.069	0.780	32.7 ± 9.1	0.428	0.179	0.185
	LM	28.7 ± 5.2**		0.059	30.4 ± 10.0		0.968	30.2 ± 5.6		0.920	33.9 ± 7.4		0.502	30.9 ± 6.3		0.258	0.166
30 min	HM	31.2 ± 6.8	0.675		27.2 ± 7.1**	0.188		32.0 ± 7.0	0.472		29.5 ± 5.9	0.100		29.4 ± 7.3	0.741		0.138
	LM	29.2 ± 6.9			29.9 ± 6.8			30.1 ± 5.0			32.4 ± 5.9			28.7 ± 6.9			0.326
NP VSL (μm/s)																		
5 min	HM	24.7 ± 8.1*** *	<0.001	0.014	22.9 ± 9.4*	0.367	0.035	21.1 ± 8.1	0.898	0.805	18.1 ± 7.4**	0.005	0.745	22.9 ± 11.8	0.299	0.123	0.122
	LM	17.1 ± 5.0*** a		0.767	20.1 ± 9.2ab		0.905	20.8 ± 7.2ab		0.749	24.4 ± 7.6*** b		0.042	19.8 ± 8.2ab		0.225	0.030
30 min	HM	19.6 ± 8.9*	0.675		18.0 ± 6.4*	0.485		21.4 ± 7.8	0.928		18.7 ± 4.6	0.645		18.2 ± 8.4	0.647		0.492
	LM	17.4 ± 6.1			19.3 ± 6.9			20.2 ± 5.9			19.6 ± 8.0*			17.1 ± 6.9			0.401
NP STR (%)																		
5 min	HM	68.4 ± 14.0* *	0.015	0.038	66.8 ± 17.5	0.186	0.406	65.3 ± 13.8	0.849	0.558	56.8 ± 18.1*	0.016	0.292	65.1 ± 21.3	0.382	0.186	0.152
	LM	57.4 ± 15.7*		0.733	61.2 ± 11.3		0.650	64.5 ± 16.4		0.728	67.7 ± 11.8* *		0.021	60.3 ± 16.4		0.413	0.140
30 min	HM	59.4 ± 15.6*	0.413		63.0 ± 14.2	0.392		63.0 ± 12.7	0.999		61.1 ± 8.0	0.420		57.6 ± 16.8	0.805		0.588
	LM	55.9 ± 14.1			59.6 ± 13.4			63.0 ± 12.0			58.3 ± 15.0*			56.4 ± 16.4			0.411
NP LIN (%)																		
5 min	HM	41.3 ± 14.4* *	0.013	0.015	39.1 ± 15.2*	0.421	0.048	35.2 ± 13.3	0.473	0.665	29.7 ± 13.8**	0.002	0.857	39.7 ± 20.4	0.265	0.081	0.073
	LM	31.7 ± 10.3*		0.640	35.1 ± 15.0		0.749	38.1 ± 14.7		0.172	41.4 ± 10.9** *		0.018	34.0 ± 14.4		0.185	0.116
30 min	HM	33.3 ± 17.0*	0.984		31.3 ± 11.6*	0.844		34.4 ± 12.9	1		30.3 ± 5.8	0.462		30.9 ± 15.0	0.358		0.794
	LM	30.3 ± 10.6			31.9 ± 10.9			33.0 ± 11.3			32.5 ± 13.5*			28.9 ± 11.9			0.734
NP WOB (%)																		
5 min	HM	56.3 ± 10.5* a	0.221	0.028	56.1 ± 10.5** ab	0.134	0.003	50.6 ± 10.8a	0.181	0.621	47.6 ± 11.6** ab	0.001	0.842	55.9 ± 14.3b	0.375	0.124	0.023
	LM	52.7 ± 9.5		0.742	51.7 ± 9.8		0.378	54.6 ± 9.9		0.055	57.2 ± 7.2**		0.052	52.6 ± 10.8		0.129	0.287
30 min	HM	52.7 ± 12.9*	0.617		46.8 ± 10.3**	0.372		52.2 ± 9.8	0.617		47.9 ± 6.6	0.104		50.1 ± 10.9	0.407		0.193
	LM	50.6 ± 10.6			49.3 ± 9.0			49.1 ± 9.6			53.1 ± 9.9			47.4 ± 11.3			0.422
MP VCL (μm/s)																		
5 min	HM	109.5 ± 22.9*** a	0.873	<0.001	123.5 ± 35.1a	0.603	0.081	149.2 ± 37.4** b	0.002	0.345	148.4 ± 28.6b	0.105	0.443	145.1 ± 26.5b	0.863	0.806	< 0.001
	LM	108.2 ± 18.3a		0.288	116.2 ± 23.5a		0.203	121.2 ± 22.3** ab		0.740	134.7 ± 30.3bc		0.672	143.6 ± 30.6c		0.343	< 0.001
30 min	HM	130.4 ± 19.9** ***	0.007		139.9 ± 29.7*	0.043		139.5 ± 34.6*	0.018		143.8 ± 31.4	0.124		143.1 ± 30.8	0.368		0.541
	LM	114.0 ± 17.7** a			124.0 ± 18.8* ab			119.0 ± 23.5* ab			131.0 ± 23.1ab			135.1 ± 30.3b			0.022
MP VAP (μm/s)																		
5 min	HM	58.7 ± 8.3*	0.028	0.881	60.0 ± 9.7	0.245	0.751	63.1 ± 7.4**	0.003	0.916	64.3 ± 9.3	0.337	0.290	64.0 ± 7.0	0.199	0.161	0.068
	LM	54.1 ± 5.8* a		0.427	57.2 ± 6.7ab		0.710	57.2 ± 5.7** ab		0.923	60.7 ± 9.4a		0.074	61.0 ± 8.9b		0.363	0.013
30 min	HM	59.1 ± 7.4**	0.005		60.8 ± 8.3*	0.048		62.9 ± 8.5**	0.005		61.0 ± 6.8*	0.016		60.8 ± 9.0	0.414		0.603
	LM	52.4 ± 7.5** a			56.5 ± 5.0* ab			57.1 ± 4.7** ab			56.3 ± 7.4* ab			58.8 ± 8.1b			0.041

Supplementary Table S2. Continued…

**MP VSL (μm/s)**																	
5 min	HM	**45.3 ± 9.7****	0.131	0.002	44.8 ± 11.1	0.162	0.248	41.0 ± 6.8	0.814	0.178	40.9 ± 10.0	0.999	0.662	41.4 ± 9.9	0.223	0.450	0.280
	LM	40.8 ± 10.7		0.209	39.6 ± 10.5		0.636	41.5 ± 7.9		0.899	40.9 ± 7.9		0.129	38.1 ± 8.5		0.567	0.737
30 min	HM	**37.2 ± 7.1**^{**a}	1		40.6 ± 8.5^{ab}	0.323		44.6 ± 10.3^{b}	0.961		40.4 ± 4.8^{ab}	0.114		39.5 ± 8.0^{ab}	0.962		**0.030**
	LM	37.2 ± 7.1			38.3 ± 6.7			41.8 ± 7.3			36.3 ± 9.3			39.6 ± 9.0			0.159
**MP STR (%)**																	
5 min	HM	**57.2 ± 11.2**^{***a}	0.582	<0.001	**54.1 ± 11.7**^{*a}	0.174	0.037	46.8 ± 9.8^{b}	0.083	0.073	47.1 ± 9.5^{b}	0.315	0.300	48.7 ± 9.3^{b}	0.651	0.999	**<0.001**
	LM	53.1 ± 11.7		0.31	52.7 ± 9.1		0.702	51.6 ± 8.3		0.563	48.6 ± 9.8		0.404	45.9 ± 7.6		0.175	0.074
30 min	HM	**47.9 ± 6.4*****	0.092		**47.2 ± 9.1***	0.914		50.1 ± 13.1	0.490		45.8 ± 6.5	0.414		45.3 ± 6.5	0.335		0.317
	LM	48.1 ± 7.5			48.3 ± 7.3			51.5 ± 8.4			46.2 ± 7.5			47.3 ± 10.3			0.182
**MP LIN (%)**																	
5 min	HM	**77.7 ± 13.2**^{***a}	0.309	<0.001	**75.9 ± 15.5**^{*a}	0.349	0.018	**65.5 ± 12.3**^{*b}	**0.032**	0.160	64.4 ± 12.0^{b}	0.357	0.410	65.2 ± 15.5^{b}	0.459	0.389	**<0.001**
	LM	75.4 ± 15.6^{a}		0.112	69.5 ± 17.0^{ab}		0.202	**71.5 ± 11.4**^{*ab}		0.853	68.2 ± 14.4^{ab}		0.599	63.3 ± 14.1^{b}		0.328	**0.046**
30 min	HM	**64.6 ± 12.0*****	0.278		**67.4 ± 12.1***	0.794		71.0 ± 12.0	0.211		67.7 ± 9.8	0.837		65.2 ± 11.7	0.328		0.268
	LM	71.0 ± 12.4			67.8 ± 10.6			73.5 ± 13.3			64.5 ± 16.3			68.5 ± 11.8			0.170
**MP WOB (%)**																	
5 min	HM	**47.1 ± 15.1**^{***a}	0.218	<0.001	**43.7 ± 16.4**^{*ab}	0.656	0.026	33.2 ± 13.0^{b}	0.064	0.357	32.3 ± 12.7^{b}	0.367	0.600	34.1 ± 14.0^{b}	0.388	0.135	**0.001**
	LM	42.5 ± 15.9^{a}		0.100	39.5 ± 14.4^{a}		0.092	39.4 ± 11.4^{a}		0.957	35.4 ± 12.8^{b}		0.477	31.3 ± 12.0^{ab}		0.734	**0.046**
30 min	HM	**32.7 ± 9.5*****	0.923		**33.8 ± 11.8***	0.664		38.1 ± 16.8	0.184		32.4 ± 8.2	0.859		31.2 ± 9.3	0.734		0.323
	LM	35.9 ± 9.7			34.7 ± 9.6			40.0 ± 12.2			31.9 ± 11.3			34.6 ± 13.0			0.251
**MP ALH (μm)**																	
5 min	HM	**2.9 ± 1.0**^{***a}	0.804	<0.001	**3.3 ± 1.3**^{*a}	0.774	0.040	**4.3 ± 1.4**^{**b}	**0.009**	0.517	4.3 ± 1.0^{b}	0.172	0.600	4.2 ± 1.0^{b}	0.872	0.744	**<0.001**
	LM	3.0 ± 0.9^{a}		0.064	3.2 ± 1.0^{ab}		0.139	**3.4 ± 0.9**^{**ab}		0.953	3.9 ± 1.1^{ab}		0.773	4.2 ± 1.0^{b}		0.323	**<0.001**
30 min	HM	**3.9 ± 0.8******	**0.032**		**4.0 ± 1.1***	0.160		4.1 ± 1.4	0.069		4.3 ± 1.0	0.327		4.3 ± 1.1	0.251		0.746
	LM	**3.4 ± 0.7***			3.6 ± 0.7			3.4 ± 0.9			4.0 ± 0.9			3.9 ± 1.1			0.131
**MP BCF (Hz)**																	
5 min	HM	**22.7 ± 4.1**^{***a}	0.716	<0.001	**23.2 ± 5.6**^{*a}	0.123	0.003	19.3 ± 4.8^{b}	0.406	0.930	18.9 ± 3.8^{b}	0.540	0.319	19.8 ± 4.0^{b}	0.118	0.142	**0.001**
	LM	**22.2 ± 4.7**^{**a}		0.007	21.0 ± 4.3^{ab}		0.147	20.3 ± 3.6^{ab}		0.524	19.6 ± 4.6^{ab}		0.066	18.1 ± 3.2^{b}		1	**0.015**
30 min	HM	**18.1 ± 3.1*****	0.925		**18.9 ± 4.1***	0.911		19.2 ± 5.1	0.282		18.0 ± 2.3	0.427		18.0 ± 4.4	1		0.725
	LM	**18.5 ± 3.8****			19.1 ± 4.3			19.7 ± 3.8			17.2 ± 4.3			18.6 ± 4.0			0.304
**RP VCL (μm/s)**																	
5 min	HM	**125.9 ± 17.7**^{**a}	0.576	0.001	**137.5 ± 16.2**^{**b}	0.718	0.006	**151.7 ± 19.4**^{***bc}	**<0.001**	0.399	151.7 ± 23.5^{c}	0.353	0.778	150.2 ± 22.7^{c}	0.089	0.401	**<0.001**
	LM	129.0 ± 17.3^{a}		0.339	135.3 ± 20.6^{ab}		0.729	**132.3 ± 15.2**^{***a}		0.163	146.2 ± 16.0^{b}		0.093	139.4 ± 20.7^{ab}		0.261	**0.024**
30 min	HM	**144.1 ± 18.7**$^{*** **}$	**<0.001**		**151.6 ± 17.6**$^{*** **}$	**<0.001**		146.6 ± 20.1	0.251		150.0 ± 21.3	0.058		155.6 ± 19.9	0.262		0.320
	LM	**122.9 ± 11.2**^{***a}			**133.3 ± 18.0**^{***ab}			139.7 ± 20.9^{b}			140.2 ± 23.3^{b}			146.1 ± 18.7^{b}			**0.005**
**RP VAP (μm/s)**																	
5 min	HM	73.3 ± 9.9	0.243	0.600	74.4 ± 10.6	0.270	0.580	**76.3 ± 9.3****	**0.004**	0.877	77.7 ± 11.2	0.201	0.884	76.8 ± 9.6	0.702	0.459	0.532
	LM	69.0 ± 13.5		0.178	71.1 ± 9.4		0.329	**69.4 ± 6.5****		0.204	73.2 ± 6.5		0.258	75.6 ± 12.6		0.537	0.275
30 min	HM	**74.8 ± 9.2*****	**<0.001**		**75.9 ± 8.5****	**0.010**		75.6 ± 8.7	0.161		**76.2 ± 10.0***	0.017		78.9 ± 9.7	0.064		0.616
	LM	**63.7 ± 8.7**^{***a}			**70.0 ± 6.4**^{**b}			72.3 ± 7.1^{b}			**70.0 ± 7.0**^{*b}			73.6 ± 9.3^{b}			**0.002**

Supplementary Table S2. Continued…

**RP VSL (µm/s)**																		
5 min	HM	67.8 ± 9.8	0.058	0.921	68.4 ± 10.5	0.141	0.765	68.4 ± 9.9	0.073	0.467	70.1 ± 11.5	0.183	0.969	69.0 ± 9.6	0.909	0.545	0.945
	LM	61.2 ± 12.0		0277	64.1 ± 9.2		0.682	62.8 ± 7.4		0.207	65.7 ± 6.2		0.398	68.7 ± 13.0		0.369	0.301
30 min	HM	**67.5 ± 8.6****	**0.001**		**67.6 ± 8.3***	**0.049**		68.0 ± 8.2	0.265		**68.7 ± 9.8***	0.025		70.7 ± 9.3	0.065		0.715
	LM	**57.1 ± 9.7**$^{**\,a}$			**63.1 ± 6.5**$^{*\,b}$			65.5 ± 7.0^{b}			**63.1 ± 6.2**$^{*\,b}$			65.7 ± 8.5^{b}			**0.006**
**RP STR (%)**																		
5 min	HM	**92.2 ± 3.3**$^{*\,a}$	0.102	0.012	**91.0 ± 2.5**$^{**\,ab}$	0.154	0.001	89.1 ± 3.1^{b}	0.292	0.444	90.0 ± 2.5^{b}	0.628	0.961	89.5 ± 2.4^{b}	0.349	0.736	**0.001**
	LM	90.3 ± 4.0		0.356	89.7 ± 3.0		0.910	90.1 ± 3.1		0.84	89.5 ± 2.4		0.869	90.3 ± 3.2		0.130	0.889
30 min	HM	**90.0 ± 2.2***	0.371		**88.7 ± 2.1****	0.154		89.7 ± 2.0	0.436		89.8 ± 1.9	0.620		89.3 ± 2.2	0.748		0.282
	LM	89.0 ± 3.8			89.9 ± 3.3			90.2 ± 2.8			89.5 ± 2.3			89.1 ± 1.6			0.604
**RP LIN (%)**																		
5 min	HM	**55.2 ± 8.9**$^{*\,a}$	0.071	0.011	**50.8 ± 7.1**$^{**\,ab}$	0.352	0.005	46.8 ± 8.4^{b}	0.190	0.377	48.1 ± 7.6^{b}	0.477	0.930	48.4 ± 7.4^{b}	0.411	0.343	**0.003**
	LM	49.7 ± 11.0		0.358	48.9 ± 7.3		0.897	49.0 ± 9.0		0.804	46.3 ± 5.6		0.423	**50.2 ± 7.9***		0.032	0.474
30 min	HM	**48.2 ± 6.4***	0.521		**45.7 ± 4.9****	0.098		47.5 ± 5.1	0.597		47.1 ± 4.4	0.975		46.6 ± 5.3	0.640		0.555
	LM	46.8 ± 6.9			48.6 ± 6.7			48.5 ± 7.1			46.7 ± 6.5			**45.9 ± 4.6***			0.566
**RP WOB (%)**																		
5 min	HM	**59.4 ± 7.6**$^{**\,a}$	0.053	0.010	**55.4 ± 6.6**$^{*\,ab}$	0.471	0.013	51.9 ± 7.5^{b}	0.204	0.304	53.1 ± 7.1^{b}	0.346	0.954	53.6 ± 6.9^{b}	0.435	0.330	**0.004**
	LM	54.4 ± 9.7		0.421	54.0 ± 6.7		0.875	53.9 ± 8.4		0.796	51.3 ± 5.1		0.350	**55.2 ± 7.1***		0.031	0.476
30 min	HM	**53.2 ± 6.0****	0.597		**51.3 ± 4.5***	0.122		52.6 ± 4.7	0.650		52.2 ± 4.1	0.877		51.9 ± 5.0	0.643		0.732
	LM	52.2 ± 5.7			53.7 ± 6.0			53.4 ± 6.2			51.9 ± 6.4			**51.2 ± 4.5***			0.589
**RP ALH (µm)**																		
5 min	HM	**3.0 ± 0.7**$^{***\,a}$	0.416	<0.001	**3.4 ± 0.7**$^{***\,b}$	0.734	<0.001	**3.9 ± 0.9**$^{*\,b}$	**0.014**	0.981	3.9 ± 0.7^{b}	0.817	0.712	**3.8 ± 0.8**$^{*\,b}$	0.078	0.021	< 0.001
	LM	3.2 ± 0.8^{a}		0.133	3.6 ± 0.9^{ab}		0.847	**3.4 ± 0.6**$^{*\,ab}$		0.144	3.9 ± 0.8^{b}		0.645	**3.4 ± 0.6**$^{**\,b}$		0.005	**0.031**
30 min	HM	**3.9 ± 0.8**$^{*\,***}$	**0.018**		**4.1 ± 0.7**$^{**\,***}$	**0.007**		3.9 ± 0.7	0.253		4.0 ± 0.6	0.060		**4.2 ± 0.6**$^{**\,*}$	**0.005**		0.311
	LM	**3.4 ± 0.4***			**3.5 ± 0.8****			3.7 ± 0.8			3.8 ± 0.9			**3.9 ± 0.7**$^{**\,**}$			0.152
**RP BCF (Hz)**																		
5 min	HM	**26.0 ± 3.4*****	0.500	<0.001	24.7 ± 4.7	0.307	0.055	23.5 ± 4.6	0.911	0.235	23.3 ± 4.1	0.327	0.265	**24.2 ± 3.8****	0.610	0.009	0.153
	LM	**25.1 ± 5.5***		0.039	23.4 ± 4.6		1	23.3 ± 3.4		0.161	23.4 ± 3.3		0.116	**24.8 ± 4.6***		0.046	0.486
30 min	HM	**21.8 ± 4.3*****	0.950		22.6 ± 2.9	0.453		22.1 ± 2.9	0.756		21.9 ± 3.7	0.882		**21.5 ± 3.1****	0.406		0.855
	LM	**21.7 ± 3.5***			23.4 ± 4.3			21.8 ± 3.9			21.8 ± 2.7			**22.3 ± 3.4***			0.531

ALH, average path velocity; BCF, beat cross frequency; CI, confidence interval; CAP, capacitating-HTF; DOPA; dopamine; HTF, human tubal fluid; HM, high motile subpopulation; LIN, linearity; LM, low motile subpopulation; MP, medium-progressive; min, minutes; NP, non-progressive; Prog, progressive; RP, rapid-progressive; SD, standard deviation; SP *p*, t-test between subpopulations; STR, straightness; TP *p*, t test between time points; VAP, average path velocity; VCL, curvilinear velocity; VSL, straight-line velocity; WOB, wobble. Values labelled in bold with an asterisks in the same column were significantly different between the HM and LM subpopulations for individual media and SCA parameters **(*p <0.05, **p <0.01 and ***p <0.001)**. Values labelled in bold and red with an asterisks in the same column were significantly different between the 5 and 30 minutes for individual subpopulations and media **(*p <0.05 and **p <0.01)**. Student's t-test was used or the Mann-Whitney test when normal distribution was absent. [a, b,c,d] Values labelled with different superscript letters in the same row were significantly different between the various media for individual subpopulations and SCA parameters. One-way ANOVA was used for parametric distributions or Kruskal-Wallis test for non-parametric distributions.

Supplementary Table S3. Comparison of mean SCA motility and kinematic parameters at 5, 30 and 60 minutes for both the low motile (LM) and high motile (HM) sperm subpopulations after treatment with HTF, CAP, 50 ng/mL, 100 ng/mL, 250 ng/mL and 500 ng/mL prolactin (mean ± SD) (n = 20).

		HTF			CAP			50 ng/mL PRL			100 ng/mL PRL			250 ng/mL PRL			500 ng/mL PRL			
		Mean ± SD	SP *p*	TP *p*	Mean ± SD	SP *p*	TP *p*	Mean ± SD	SP *p*	TP *p*	Mean ± SD	SP *p*	TP *p*	Mean ± SD	SP *p*	TP *p*	Mean ± SD	SP *p*	TP *p*	ANOVA
**Prog (%)**																				
5 min	HM	33.3 ± 21.1***	<0.001	0.184	42.2 ± 21.5***	<0.001	0.515	39.5 ± 20.3**	0.002	0.625	36.9 ± 19.7*	0.011	0.710	38.2 ± 19.1**	0.001	0.801	36.3 ± 21.4***	<0.001	0.524	0.757
	LM	10.0 ± 9.8^{***a}		0.542	15.0 ± 11.8^{***ab}		0.641	22.7 ± 14.1^{**b}		0.779	23.4 ± 15.3^{*b}		0.435	20.9 ± 15.4^{**b}		0.752	17.9 ± 13.6^{***ab}		0.711	0.005
30 min	HM	27.7 ± 19.5***	<0.001		37.0 ± 22.2**	0.008		37.5 ± 19.1**	0.003		32.6 ± 19.3	0.241		34.6 ± 20.6	0.054		34.5 ± 23.2**	0.002		0.636
	LM	9.4 ± 9.7^{***a}			19.0 ± 17.4^{**ab}			21.5 ± 19.5^{**ab}			25.9 ± 19.9^{b}			23.3 ± 20.4^{ab}			16.3 ± 13.9^{**ab}			0.024
60 min	HM	22.1 ± 17.7**	0.002		34.8 ± 21.7**	0.004		34.0 ± 15.0**	0.006		33.3 ± 17.0**	0.009		35.9 ± 15.6**	0.003		29.1 ± 18.0*	0.011		0.155
	LM	7.0 ± 8.2^{**a}			16.8 ± 13.1^{**ab}			19.2 ± 13.2^{**ab}			19.2 ± 14.6^{**b}			19.3 ± 16.6^{**ab}			14.4 ± 14.7^{*ab}			0.044
**Motile (%)**																				
5 min	HM	61.5 ± 23.5***	<0.001	0.128	68.7 ± 22.0***	<0.001	0.952	67.4 ± 21.1***	<0.001	0.842	65.9 ± 22.4**	0.001	0.998	68.7 ± 21.6***	<0.001	0.613	65.2 ± 22.2***	<0.001	0.656	0.873
	LM	28.8 ± 19.5***		0.364	39.1 ± 19.8***		0.822	42.7 ± 19.4***		0.738	44.5 ± 20.6**		0.370	40.8 ± 20.2***		0.783	36.2 ± 15.7***		0.541	0.077
30 min	HM	56.0 ± 23.7***	<0.001		67.1 ± 21.9***	<0.001		66.0 ± 22.4***	<0.001		66.3 ± 20.9***	<0.001		63.0 ± 24.6**	0.002		66.8 ± 23.8***	<0.001		0.544
	LM	26.2 ± 18.8***			38.1 ± 20.8***			38.3 ± 21.1***			43.3 ± 21.5***			40.9 ± 20.5**			38.1 ± 15.6***			0.072
60 min	HM	47.2 ± 20.1^{***a}	<0.001		66.9 ± 20.3^{***b}	<0.001		63.8 ± 16.6^{***b}	<0.001		66.3 ± 18.9^{***b}	<0.001		63.2 ± 19.4^{***b}	<0.001		59.2 ± 20.7^{***ab}	<0.001		0.024
	LM	20.7 ± 17.3^{***a}			35.4 ± 18.6^{***b}			40.0 ± 16.7^{***b}			36.5 ± 15.2^{***b}			37.2 ± 16.3^{***b}			32.8 ± 15.2^{***b}			0.010
**RP (%)**																				
5 min	HM	9.8 ± 10.4**	0.004	0.626	11.5 ± 14.8	0.133	0.999	11.8 ± 12.3*	0.033	0.962	9.3 ± 11.2	0.144	0.911	11.1 ± 11.6*	0.027	0.870	10.3 ± 12.8	0.169	0.869	0.979
	LM	2.2 ± 3.7**		0.994	4.3 ± 6.0		0.861	4.3 ± 5.0*		0.370	4.8 ± 7.1		0.467	5.6 ± 9.3*		0.788	4.5 ± 5.9		0.845	0.775
30 min	HM	7.8 ± 9.4	0.065		11.6 ± 14.4	0.142		12.8 ± 13.5	0.155		10.5 ± 12.8	0.369		12.6 ± 13.5	0.139		12.1 ± 13.9*	0.023		0.782
	LM	2.1 ± 3.2			5.4 ± 8.5			7.3 ± 11.6			7.6 ± 10.7			7.5 ± 11.2			4.1 ± 6.2*			0.596
60 min	HM	7.2 ± 8.2	0.115		11.6 ± 14.5	0.335		11.9 ± 13.9	0.606		9.1 ± 10.2	0.091		10.9 ± 10.3*	0.049		10.4 ± 12.6	0.118		0.874
	LM	2.1 ± 2.9			5.4 ± 7.3			7.8 ± 9.2			4.8 ± 7.5			6.1 ± 8.2*			5.4 ± 10.6			0.489
**MP (%)**																				
5 min	HM	23.5 ± 15.3***	<0.001	0.128	30.7 ± 14.6***	<0.001	0.192	27.7 ± 13.8*	0.019	0.312	27.5 ± 14.5*	0.021	0.381	25.7 ± 11.7**	0.004	0.521	25.9 ± 12.0***	<0.001	0.138	0.577
	LM	7.8 ± 9.2^{***a}		0.447	10.7 ± 10.3^{***ab}		0.617	18.4 ± 12.8^{*b}		0.253	18.6 ± 12.2^{*b}		0.539	15.3 ± 11.7^{***ab}		0.766	13.4 ± 12.2^{***ab}		0.425	0.007
30 min	HM	19.9 ± 12.2***	<0.001		25.4 ± 14.4**	0.005		24.3 ± 11.2**	0.003		22.2 ± 12.2	0.330		22.0 ± 12.0	0.101		22.4 ± 13.2**	0.008		0.748
	LM	7.3 ± 7.8***			13.6 ± 12.7**			14.2 ± 12.1**			18.3 ± 14.8			15.9 ± 13.3			12.2 ± 10.9**			0.057
60 min	HM	14.9 ± 12.8**	0.002		23.2 ± 12.6**	0.001		22.1 ± 11.0**	0.002		24.2 ± 13.4*	0.025		25.0 ± 11.3**	0.003		18.7 ± 8.3**	0.004		0.075
	LM	4.9 ± 5.9**			11.4 ± 7.5**			11.4 ± 7.9**			14.3 ± 13.8*			13.2 ± 11.2**			8.9 ± 10.6**			0.055
**NP (%)**																				
5 min	HM	28.2 ± 19.1*	0.048	0.793	26.5 ± 20.9	0.805	0.602	27.9 ± 16.1	0.084	0.950	29.1 ± 19.2	0.146	0.716	30.6 ± 21.1	0.108	0.869	29.0 ± 19.6	0.087	0.885	0.988
	LM	18.8 ± 15.2*		0.481	24.1 ± 17.7		0.461	20.0 ± 14.7		0.619	21.1 ± 15.4		0.551	19.8 ± 12.3		0.801	18.3 ± 13.6		0.617	0.807
30 min	HM	28.3 ± 16.0**	0.007		30.1 ± 18.2	0.053		28.5 ± 19.7*	0.026		33.7 ± 23.6*	0.014		28.4 ± 23.2	0.149		32.2 ± 27.0	0.327		0.950
	LM	16.8 ± 13.3**			19.9 ± 12.5			16.9 ± 14.5*			17.4 ± 13.1*			17.5 ± 13.7			21.8 ± 15.7			0.766
60 min	HM	25.1 ± 15.6*	0.014		32.1 ± 15.2**	0.004		29.7 ± 20.5	0.300		33.0 ± 19.0**	0.003		27.3 ± 17.3	0.062		30.1 ± 21.3*	0.049		0.780
	LM	13.7 ± 11.7*			18.6 ± 14.4**			20.8 ± 13.3			17.3 ± 10.1**			17.9 ± 12.6			18.5 ± 10.9*			0.631
**Rapid (%)**																				
5 min	HM	15.9 ± 18.0*	0.025	0.570	21.1 ± 24.2	0.152	0.943	20.4 ± 21.2	0.060	0.725	16.9 ± 20.5	0.485	0.863	21.0 ± 23.9*	0.020	0.752	19.5 ± 26.0	0.416	0.769	0.946
	LM	4.7 ± 6.2*		0.804	7.4 ± 8.5		0.870	10.0 ± 12.9		0.935	11.5 ± 15.0		0.513	9.0 ± 12.8*		0.718	8.8 ± 10.1		0.990	0.694
30 min	HM	13.5 ± 15.6*	0.029		19.8 ± 25.9	0.172		21.8 ± 21.9	0.118		17.3 ± 21.7	0.707		20.4 ± 20.1	0.090		20.3 ± 23.6	0.159		0.816
	LM	4.4 ± 6.9*			9.0 ± 13.4			11.4 ± 15.7			13.4 ± 16.9			12.1 ± 16.9			8.8 ± 9.8			0.390
60 min	HM	10.8 ± 12.2	0.063		18.6 ± 22.6	0.104		16.7 ± 19.1	0.403		14.1 ± 18.1	0.425		16.4 ± 18.6	0.166		15.3 ± 20.2	0.217		0.856
	LM	3.5 ± 4.4			7.6 ± 11.0			10.5 ± 13.0			8.2 ± 10.8			9.6 ± 11.1			9.2 ± 13.1			0.626

Supplementary Table S3. Continued…

**Medium (%)**																				
**5 min**	**HM**	**22.9 ± 16.5*****	**<0.001**	0.279	**27.1 ± 20.2****	**0.005**	0.850	26.4 ± 19.0	0.064	0.798	27.2 ± 20.5	0.065	0.546	24.2 ± 17.8	0.108	0.651	**23.5 ± 17.1***	**0.018**	0.745	0.937
	**LM**	**8.0 ± 9.7*****		0.574	**11.3 ± 11.5****		0.784	16.4 ± 15.4		0.780	15.8 ± 13.9		0.865	15.4 ± 14.5		0.704	**12.2 ± 11.3***		0.267	0.177
**30 min**	**HM**	**19.8 ± 13.3*****	**<0.001**		24.0 ± 19.8	0.072		22.9 ± 19.0	0.110		21.5 ± 16.7	0.194		23.0 ± 19.1	0.170		**20.3 ± 15.5***	**0.041**		0.998
	**LM**	**6.7 ± 8.0*****			13.5 ± 14.0			14.3 ± 12.8			15.7 ± 14.5			15.8 ± 15.4			**12.5 ± 9.4***			0.121
**60 min**	**HM**	**15.6 ± 13.8*****	**<0.001**		**25.1 ± 15.6****	**0.008**		**23.6 ± 16.8***	**0.038**		**26.1 ± 19.1***	**0.017**		**28.3 ± 20.9****	**0.007**		**20.6 ± 13.5****	**0.001**		0.239
	**LM**	**5.2 ± 8.6^{***a}**			**13.3 ± 9.5^{**b}**			**13.8 ± 10.8^{*b}**			**13.6 ± 15.6^{*ab}**			**12.4 ± 12.1^{**b}**			**8.0 ± 8.1^{**ab}**			**0.05**
**Slow (%)**																				
**5 min**	**HM**	22.7 ± 16.7	0.083	0.896	20.5 ± 17.0	0.926	0.776	20.7 ± 13.5	0.298	0.857	21.9 ± 15.4	0.224	0.559	23.5 ± 16.9	0.080	0.572	22.2 ± 15.1	0.076	0.761	0.985
	**LM**	16.1 ± 13.2		0.515	20.4 ± 16.0		0.311	16.3 ± 12.2		0.618	17.2 ± 13.5		0.617	16.4 ± 9.7		0.515	15.2 ± 11.3		0.875	0.792
**30 min**	**HM**	**22.7 ± 13.8***	**0.047**		23.2 ± 15.0	0.056		21.3 ± 17.9	0.083		**27.5 ± 21.5***	**0.019**		19.6 ± 18.4	0.415		26.2 ± 23.0	0.261		0.676
	**LM**	**15.2 ± 12.3***			15.6 ± 11.8			13.0 ± 11.5			**14.1 ± 10.4***			12.9 ± 10.2			16.8 ± 12.6			0.827
**60 min**	**HM**	**20.8 ± 13.7***	**0.037**		**23.2 ± 12.4***	**0.029**		23.4 ± 17.9	0.108		**26.0 ± 18.1***	**0.019**		18.5 ± 13.2	0.422		23.3 ± 18.7	0.405		0.772
	**LM**	**12.0 ± 9.3***			**14.4 ± 12.4***			15.8 ± 13.2			**14.6 ± 9.0***			15.2 ± 11.6			15.6 ± 9.2			0.912
**Motile VCL (μm/s)**																				
**5 min**	**HM**	111.8 ± 17.3	0.086	0.825	124.5 ± 22.1	0.144	0.998	125.2 ± 20.5	0.533	0.182	122.6 ± 22.0	0.920	0.899	**125.9 ± 22.2***	**0.026**	**0.638**	126.1 ± 26.0	0.196	0.674	0.189
	**LM**	102.4 ± 19.6		0.243	113.7 ± 28.0		0.923	121.0 ± 26.3		0.607	121.9 ± 26.7		0.885	**111.4 ± 21.4***		0.546	116.7 ± 23.6		0.974	0.074
**30 min**	**HM**	**108.8 ± 17.8^{**a}**	**0.007**		124.8 ± 23.7^{b}	0.148		**135.6 ± 20.2^{**b}**	**0.002**		121.3 ± 20.1^{b}	0.812		**132.0 ± 22.7^{*b}**	**0.040**		127.2 ± 22.4^{b}	0.164		**0.001**
	**LM**	**92.8 ± 20.3^{**a}**			114.9 ± 21.6^{b}			**118.1 ± 16.9^{**b}**			119.6 ± 30.5^{b}			**118.6 ± 21.1^{*b}**			116.5 ± 29.5^{b}			**0.001**
**60 min**	**HM**	**111.2 ± 17.9^{*a}**	**0.017**		125.0 ± 20.4^{ab}	0.052		130.7 ± 15.7^{b}	0.429		124.2 ± 17.9^{ab}	0.440		130.7 ± 25.6^{b}	0.054		121.1 ± 20.5^{ab}	0.476		**0.034**
	**LM**	**97.7 ± 17.5***			111.9 ± 22.3			125.2 ± 25.7			117.3 ± 34.2			113.8 ± 26.7			114.8 ± 31.9			0.092
**Motile VAP (μm/s)**																				
**5 min**	**HM**	57.6 ± 9.0	0.067	0.661	60.1 ± 10.4	0.343	0.855	61.2 ± 9.6	0.217	0.535	59.2 ± 9.9	0.691	0.791	**60.0 ± 8.6***	**0.017**	0.370	59.5 ± 10.2	0.167	0.991	0.872
	**LM**	52.8 ± 8.7		0.199	55.3 ± 10.6		0.798	58.0 ± 7.8		0.280	58.1 ± 10.0		0.863	**54.2 ± 7.8***		0.163	56.4 ± 9.3		0.856	0.275
**30 min**	**HM**	**54.9 ± 11.1^{*a}**	**0.026**		58.6 ± 10.0^{ab}	0.563		63.3 ± 8.1^{b}	0.073		58.0 ± 8.1^{ab}	0.535		63.6 ± 9.8^{b}	0.103		59.8 ± 8.5^{ab}	0.055		**0.014**
	**LM**	**48.2 ± 8.3^{*a}**			57.1 ± 8.2^{b}			59.3 ± 6.8^{b}			60.0 ± 13.4^{b}			59.2 ± 8.2^{b}			55.0 ± 11.6^{b}			**< 0.001**
**60 min**	**HM**	57.0 ± 12.1	0.118		60.3 ± 11.8	0.362		64.0 ± 8.8	0.502		59.9 ± 10.0	0.741		63.7 ± 11.5	0.148		59.4 ± 10.4	0.213		0.329
	**LM**	51.3 ± 9.3			56.9 ± 10.7			62.9 ± 14.9			58.5 ± 14.7			58.6 ± 12.8			54.5 ± 13.3			0.140
**Motile VSL (μm/s)**																				
**5 min**	**HM**	45.1 ± 11.9	0.118	0.498	47.3 ± 11.0	0.248	0.690	48.1 ± 10.2	0.094	0.681	46.0 ± 9.0	0.223	0.690	**46.7 ± 6.7***	**0.032**	0.542	45.7 ± 9.7	0.171	0.970	0.908
	**LM**	40.0 ± 10.3		0.458	42.2 ± 10.3		0.690	43.3 ± 9.3		0.270	43.1 ± 7.1		0.416	**41.8 ± 8.3***		0.336	41.5 ± 11.2		0.547	0.854
**30 min**	**HM**	40.9 ± 12.9	0.172		44.5 ± 9.6	0.267		47.9 ± 8.2	0.368		44.4 ± 8.4	0.567		48.2 ± 11.6	0.494		**45.5 ± 8.9***	**0.013**		0.142
	**LM**	36.1 ± 10.0^{a}			42.6 ± 10.4^{ab}			45.6 ± 9.4^{b}			46.3 ± 13.8^{b}			46.0 ± 10.9^{b}			**38.5 ± 9.9^{*ab}**			**0.004**
**60 min**	**HM**	43.5 ± 11.7	0.166		45.8 ± 11.9	0.682		50.3 ± 9.9	0.431		46.7 ± 10.5	0.965		50.3 ± 11.1	0.338		**46.2 ± 11.1***	**0.036**		0.359
	**LM**	37.8 ± 11.0			44.3 ± 10.3			49.1 ± 15.9			43.1 ± 14.4			46.3 ± 14.3			**38.8 ± 10.0***			0.073

Supplementary Table S3. Continued…

**Motile STR (%)**																				
**5 min**	**HM**	75.3 ± 10.9	0.343	0.330	77.2 ± 7.9	0.147	0.180	76.8 ± 7.0	0.082	0.340	75.9 ± 6.9	0.126	0.543	76.2 ± 6.4	0.306	0.524	74.6 ± 7.8	0.523	0.807	0.887
	**LM**	73.2 ± 11.9		0.597	73.6 ± 8.7		0.642	72.5 ± 9.7		0.632	72.8 ± 6.8		0.476	74.0 ± 8.2		0.761	71.9 ± 11.9		0.500	0.979
**30 min**	**HM**	70.8 ± 11.0	0.921		73.7 ± 7.2	0.307		73.8 ± 7.5	0.370		73.7 ± 7.1	0.974		74.0 ± 9.4	0.387		73.3 ± 6.8	0.070		0.784
	**LM**	70.4 ± 13.2			71.7 ± 9.9			74.8 ± 9.8			73.8 ± 9.8			75.8 ± 10.1			68.4 ± 11.2			0.150
**60 min**	**HM**	73.0 ± 8.0	0.166		73.2 ± 8.2	0.770		75.8 ± 6.9	0.678		74.5 ± 6.2	0.405		76.2 ± 6.2	0.917		74.3 ± 6.9	0.088		0.668
	**LM**	69.5 ± 11.2			74.1 ± 7.3			74.8 ± 8.3			70.4 ± 10.6			75.9 ± 11.1			68.9 ± 10.0			0.132
**Motile LIN (%)**																				
**5 min**	**HM**	42.4 ± 11.2	0.769	0.446	41.4 ± 10.6	0.574	0.357	40.9 ± 10.4	0.550	0.632	40.0 ± 7.4	0.363	0.558	40.1 ± 7.1	0.724	0.758	38.8 ± 7.4	0.613	0.711	0.820
	**LM**	41.4 ± 11.0		0.551	39.8 ± 8.3		0.892	38.2 ± 7.7		0.522	37.9 ± 8.2		0.533	39.4 ± 6.5		0.423	37.6 ± 8.9		0.568	0.636
**30 min**	**HM**	38.7 ± 11.0	0.475		37.2 ± 5.7	0.434		37.9 ± 8.6	0.265		38.2 ± 7.6	0.433		38.5 ± 8.5	0.269		37.5 ± 6.7	0.405		0.987
	**LM**	39.2 ± 9.8			39.0 ± 9.5			40.6 ± 8.0			40.4 ± 10.0			41.4 ± 9.2			35.6 ± 8.6			0.321
**60 min**	**HM**	39.6 ± 7.2	0.328		38.2 ± 7.9	0.420		39.9 ± 7.7	0.943		37.9 ± 5.9	0.946		39.7 ± 6.8	0.274		39.0 ± 6.1	0.060		0.927
	**LM**	38.3 ± 7.1			40.2 ± 7.2			40.1 ± 7.2			37.8 ± 8.2			42.6 ± 8.9			35.2 ± 6.2			0.060
**Motile WOB (%)**																				
**5 min**	**HM**	54.1 ± 8.4	0.885	0.704	51.3 ± 9.1	0.699	0.636	51.2 ± 8.9	0.665	0.649	51.1 ± 6.4	0.906	0.648	50.6 ± 6.0	0.733	1	49.9 ± 5.9	0.636	0.655	0.488
	**LM**	54.5 ± 7.8		0.878	52.3 ± 7.4		0.889	51.2 ± 6.5		0.631	50.8 ± 7.2		0.555	51.2 ± 5.5		0.247	50.7 ± 5.7		0.830	0.365
**30 min**	**HM**	52.4 ± 7.9	0.574		**49.3 ± 6.0***	**0.048**		49.3 ± 7.3	0.077		49.9 ± 6.6	0.228		50.6 ± 6.7	0.228		49.4 ± 5.6	0.459		0.599
	**LM**	53.6 ± 7.0			**53.3 ± 7.4***			52.9 ± 6.2			53.0 ± 7.5			52.9 ± 6.4			50.7 ± 6.4			0.731
**60 min**	**HM**	52.7 ± 6.0	0.704		50.3 ± 7.0	0.254		51.0 ± 6.3	0.678		49.4 ± 5.2	0.226		50.6 ± 6.1	0.083		50.9 ± 5.2	0.466		0.661
	**LM**	53.5 ± 4.9			53.0 ± 7.4			51.8 ± 5.5			52.3 ± 6.3			54.4 ± 7.2			49.7 ± 5.2			0.263
**Motile ALH (μm)**																				
**5 min**	**HM**	3.1 ± 0.6	0.883	0.834	3.4 ± 0.8	0.495	0.796	3.4 ± 0.8	0.514	0.104	3.4 ± 0.7	0.539	0.619	3.5 ± 0.8	0.216	0.424	3.5 ± 0.8	0.596	0.676	0.335
	**LM**	3.0 ± 0.7		0.152	3.3 ± 0.8		0.752	3.6 ± 0.9		0.573	3.5 ± 0.8		0.877	3.2 ± 0.6		0.499	3.4 ± 0.7		0.973	0.131
**30 min**	**HM**	**3.1 ± 0.5^{*a}**	**0.027**		3.6 ± 0.7^{ab}	0.236		**3.8 ± 0.7^{*b}**	**0.023**		3.5 ± 0.6^{b}	0.639		3.7 ± 0.7^{b}	0.129		3.7 ± 0.7^{b}	0.382		**0.005**
	**LM**	**2.7 ± 0.6^{*a}**			3.4 ± 0.7^{b}			**3.4 ± 0.6^{*b}**			3.4 ± 0.9^{b}			3.4 ± 0.7^{b}			3.5 ± 0.9^{b}			**0.006**
**60 min**	**HM**	3.2 ± 0.5	0.054		3.5 ± 0.6	0.088		3.7 ± 0.5	0.615		3.6 ± 0.5	0.707		3.7 ± 0.7	0.164		3.5 ± 0.5	0.963		0.057
	**LM**	2.9 ± 0.3^{a}			3.2 ± 0.6^{ab}			3.6 ± 0.6^{b}			3.5 ± 1.0^{b}			3.3 ± 0.7^{b}			3.5 ± 0.8^{b}			**0.029**
**Motile BCF (Hz)**																				
**5 min**	**HM**	**22.8 ± 4.3***	**0.01**	0.144	**23.1 ± 3.6***	**0.028**	0.090	**22.9 ± 3.6****	**0.002**	0.075	**22.5 ± 2.5^{**a}**	**0.002**	0.053	**22.9 ± 3.3***	**0.026**	0.124	**21.8 ± 2.8***	**0.028**	0.408	0.799
	**LM**	**20.2 ± 3.9***		0.625	**20.9 ± 2.4***		0.588	**19.7 ± 2.7****		0.311	**19.7 ± 3.2****		0.405	**20.9 ± 2.8***		0.595	**19.9 ± 2.8***		0.074	0.577
**30 min**	**HM**	20.7 ± 4.0	0.240		21.1 ± 2.6	0.341		20.9 ± 2.9	0.773		**20.9 ± 2.8^{ab}**	0.268		21.1 ± 3.2	0.331		**20.9 ± 2.5****	**0.003**		0.998
	**LM**	19.7 ± 3.9			20.2 ± 3.7			20.8 ± 3.3			19.9 ± 3.0			20.2 ± 3.1			**18.1 ± 3.4****			0.129
**60 min**	**HM**	20.7 ± 3.7	0.088		21.4 ± 3.2	0.469		**21.3 ± 2.7***	**0.024**		**20.9 ± 2.0^{*b}**	**0.011**		**21.8 ± 2.5***	**0.049**		**21.0 ± 1.9*****	**<0.001**		0.838
	**LM**	18.9 ± 4.0			20.7 ± 3.4			**19.6 ± 2.8***			**18.7 ± 3.0***			**20.0 ± 2.8***			**17.7 ± 3.1*****			0.075

Supplementary Table S3. Continued…

**NP VCL (μm/s)**																				
**5 min**	**HM**	85.7 ± 22.4	0.174	0.850	96.3 ± 33.2	0.564	0.679	94.8 ± 34.2	0.524	0.874	91.6 ± 31.9	0.606	0.638	95.3 ± 29.8	0.322	0.658	96.5 ± 35.9	0.303	0.749	0.822
	**LM**	78.8 ± 23.5		0.674	91.0 ± 31.6		0.892	89.9 ± 36.8		0.545	87.6 ± 33.6		0.980	86.8 ± 26.4		0.809	86.5 ± 30.9		0.917	0.801
**30 min**	**HM**	85.8 ± 25.1	0.066		95.5 ± 32.5	0.475		99.3 ± 41.3	0.742		91.7 ± 29.3	0.431		98.2 ± 33.2	0.392		89.8 ± 27.0	0.949		0.778
	**LM**	73.2 ± 19.9			87.1 ± 29.8			91.2 ± 34.2			85.8 ± 36.1			89.8 ± 27.4			90.3 ± 31.0			0.415
**60 min**	**HM**	89.3 ± 20.4	0.054		103.5 ± 28.3	0.087		94.4 ± 28.5	0.502		99.3 ± 27.2	0.184		102.7 ± 32.6	0.188		96.7 ± 26.5	0.164		0.608
	**LM**	75.6 ± 20.7			88.3 ± 23.8			101.2 ± 32.2			86.0 ± 33.1			89.4 ± 27.3			84.5 ± 26.5			0.174
**NP VAP (μm/s)**																				
**5 min**	**HM**	41.1 ± 11.3	0.773	0.858	42.7 ± 13.6	0.790	0.800	42.8 ± 13.8	0.629	0.933	42.1 ± 14.4	0.660	0.694	43.7 ± 11.9	0.242	0.911	41.8 ± 13.9	0.711	0.795	0.988
	**LM**	40.6 ± 10.5		0.613	43.6 ± 10.8		0.870	41.3 ± 13.5		0.521	40.4 ± 12.3		0.949	39.8 ± 11.0		0.469	41.3 ± 12.3		0.621	0.909
**30 min**	**HM**	40.3 ± 12.2	0.419		42.0 ± 13.7	0.967		41.7 ± 15.7	0.853		40.2 ± 14.0	0.882		43.8 ± 14.0	0.645		40.8 ± 12.1	0.832		0.954
	**LM**	37.4 ± 11.8			42.2 ± 11.0			44.0 ± 11.0			40.4 ± 13.7			43.0 ± 10.2			41.5 ± 10.0			0.448
**60 min**	**HM**	42.3 ± 11.5	0.274		44.8 ± 13.7	0.197		43.2 ± 13.9	0.574		44.0 ± 14.1	0.287		45.3 ± 14.3	0.504		43.5 ± 12.5	0.215		0.986
	**LM**	38.7 ± 11.0			41.9 ± 13.0			45.7 ± 11.9			39.2 ± 14.4			43.8 ± 13.0			38.2 ± 14.3			0.419
**NP VSL (μm/s)**																				
**5 min**	**HM**	27.6 ± 8.8	0.759	0.707	29.3 ± 8.5	0.949	0.632	29.6 ± 9.0	0.344	0.636	28.1 ± 9.3	0.232	0.715	30.5 ± 8.0	0.143	0.767	27.7 ± 9.1	0.951	0.737	0.827
	**LM**	28.5 ± 10.3		0.538	29.1 ± 10.2		0.632	27.1 ± 9.4		0.499	25.7 ± 9.2		0.788	26.9 ± 8.9		0.509	27.2 ± 10.0		0.865	0.862
**30 min**	**HM**	25.9 ± 9.5	0.848		26.8 ± 9.7	0.726		27.0 ± 10.8	0.503		26.8 ± 10.4	0.952		28.7 ± 9.7	0.995		26.2 ± 9.4	0.718		0.952
	**LM**	25.2 ± 11.8			27.8 ± 8.9			30.1 ± 8.2			27.0 ± 11.0			28.7 ± 11.0			26.6 ± 6.8			0.720
**60 min**	**HM**	27.9 ± 8.3	0.438		29.0 ± 10.0	0.339		28.9 ± 10.0	0.964		29.4 ± 10.7	0.198		30.3 ± 9.8	0.988		28.3 ± 9.3	0.287		0.984
	**LM**	25.6 ± 10.1			26.3 ± 9.2			29.5 ± 8.7			24.8 ± 10.7			30.9 ± 13.6			24.7 ± 11.0			0.373
**NP STR (%)**																				
**5 min**	**HM**	66.0 ± 8.8	0.578	0.558	**68.9 ± 9.0**[a]	0.392	0.050	68.8 ± 8.2	0.286	0.095	66.0 ± 7.8	0.259	0.557	69.5 ± 9.0	0.097	0.145	66.2 ± 8.3	0.736	0.357	0.474
	**LM**	67.9 ± 13.7		0.348	66.0 ± 13.8		0.379	64.3 ± 10.2		0.408	62.5 ± 12.8		0.781	64.6 ± 11.0		0.827	65.2 ± 12.4		0.457	0.769
**30 min**	**HM**	63.3 ± 8.4	0.517		**63.4 ± 7.8**[ab]	0.686		63.0 ± 10.2	0.163		64.1 ± 8.0	0.969		65.4 ± 8.1	0.775		62.2 ± 13.5	0.915		0.912
	**LM**	61.4 ± 19.5			64.6 ± 12.3			67.8 ± 12.9			64.0 ± 10.1			65.1 ± 15.6			63.3 ± 8.5			0.849
**60 min**	**HM**	65.1 ± 8.7	0.565		**63.5 ± 8.6**[b]	0.339		65.6 ± 8.3	0.489		63.4 ± 8.1	0.548		65.3 ± 7.2	0.631		63.0 ± 7.1	0.512		0.872
	**LM**	63.0 ± 12.7			60.5 ± 12.2			63.3 ± 11.1			61.7 ± 9.5			67.2 ± 15.3			61.0 ± 11.0			0.602
**NP LIN (%)**																				
**5 min**	**HM**	36.2 ± 13.5	0.209	0.281	33.9 ± 7.1	0.657	0.061	34.0 ± 8.8	0.775	0.219	32.8 ± 7.8	1	0.408	35.8 ± 9.4	0.592	0.127	31.8 ± 5.8	0.357	0.877	0.508
	**LM**	38.5 ± 12.1		0.296	35.2 ± 12.2		0.459	33.6 ± 7.9		0.244	32.0 ± 11.4		0.701	32.8 ± 8.4		0.587	33.9 ± 9.6		0.257	0.340
**30 min**	**HM**	31.9 ± 7.7	0.602		30.1 ± 5.3	0.093		**29.6 ± 9.7***	**0.025**		30.2 ± 8.5	0.324		32.0 ± 8.9	0.173		30.9 ± 10.4	0.629		0.911
	**LM**	33.6 ± 13.4			35.4 ± 13.8			**36.3 ± 10.1***			32.8 ± 8.9			34.8 ± 11.6			32.2 ± 7.6			0.795
**60 min**	**HM**	32.5 ± 6.5	0.641		29.9 ± 6.4	0.675		33.3 ± 8.6	0.539		29.9 ± 7.2	0.942		30.8 ± 7.0	0.106		30.7 ± 5.9	0.608		0.579
	**LM**	33.7 ± 8.9			31.1 ± 9.4			31.5 ± 8.8			30.4 ± 6.4			36.2 ± 12.5			29.3 ± 10.0			0.270

Supplementary Table S3. Continued…

**NP WOB (%)**																				
5 min	**HM**	50.5 ± 7.4	0.116	0.695	47.3 ± 8.3	0.111	0.465	47.6 ± 8.3	0.333	0.312	47.8 ± 8.0	0.496	0.551	49.3 ± 7.5	0.951	0.343	46.4 ± 6.2	0.060	0.895	0.477
	**LM**	54.3 ± 8.8		0.463	51.8 ± 10.8		0.615	49.9 ± 7.3		0.279	49.7 ± 10.2		0.712	48.1 ± 8.8		0.389	50.3 ± 7.9		0.269	0.262
30 min	**HM**	49.1 ± 7.2	0.366		**46.5 ± 7.7***	**0.033**		**44.8 ± 9.4****	**0.009**		45.5 ± 9.9	0.112		47.5 ± 9.0	0.201		47.3 ± 8.3	0.322		0.587
	**LM**	51.4 ± 9.7			**53.1 ± 12.3***			**52.0 ± 9.0****			49.8 ± 8.5			50.8 ± 7.9			49.7 ± 8.1			0.810
60 min	**HM**	48.9 ± 5.9	0.247		45.4 ± 6.7	0.109		48.5 ± 7.6	0.828		45.3 ± 7.9	0.431		**45.7 ± 7.1***	**0.039**		47.0 ± 6.1	0.850		0.407
	**LM**	51.5 ± 7.3			49.7 ± 9.2			48.0 ± 7.5			47.8 ± 7.4			**51.6 ± 9.6***			46.0 ± 11.4			0.338
**MP VCL (µm/s)**																				
5 min	**HM**	122.6 ± 23.6	0.29	0.531	128.7 ± 27.9	0.757	0.314	135.7 ± 21.9	0.699	0.107	134.0 ± 26.2	0.378	0.685	135.2 ± 24.7	0.121	0.444	133.5 ± 25.3	0.879	0.373	0.338
	**LM**	130.5 ± 22.6		0.087	131.6 ± 31.8		0.282	133.0 ± 25.5		0.656	141.3 ± 29.9		0.919	123.9 ± 23.1		0.419	134.8 ± 29.0		0.069	0.438
30 min	**HM**	125.3 ± 17.4^{a}	0.081		139.5 ± 21.8^{ab}	0.359		**149.5 ± 26.5**$^{**\,b}$	**0.003**		135.5 ± 21.8^{ab}	0.693		143.9 ± 24.9^{ab}	0.152		140.3 ± 26.4^{ab}	0.484		**0.020**
	**LM**	114.4 ± 20.4			133.6 ± 17.5			**126.3 ± 22.5****			138.5 ± 28.1			125.9 ± 35.1			133.6 ± 34.8			0.121
60 min	**HM**	129.7 ± 20.0	0.930		136.0 ± 21.4	0.061		**146.4 ± 20.3***	**0.033**		139.8 ± 12.1	0.760		140.5 ± 19.1	0.533		**129.9 ± 20.3***	**0.026**		0.061
	**LM**	129.0 ± 24.7^{ac}			123.0 ± 17.4^{c}			**129.1 ± 25.7**$^{*\,ac}$			142.2 ± 30.6^{ab}			135.9 ± 23.0^{abc}			**160.3 ± 38.6**^{*b}			**0.030**
**MP VAP (µm/s)**																				
5 min	**HM**	65.7 ± 9.6	0.902	0.538	66.1 ± 7.9	0.667	0.720	67.9 ± 8.3	0.535	0.321	68.0 ± 8.2	0.530	0.669	67.5 ± 10.3	0.321	0.386	67.8 ± 7.9	0.651	0.908	0.899
	**LM**	67.3 ± 9.6		0.082	64.9 ± 10.6		0.910	66.3 ± 9.6		0.925	69.8 ± 10.7		0.325	64.7 ± 7.7		0.374	66.6 ± 9.9		0.605	0.548
30 min	**HM**	64.6 ± 13.5	0.283		67.1 ± 10.0	0.724		70.1 ± 7.3	0.113		67.4 ± 8.0	0.966		71.2 ± 10.4	0.294		66.9 ± 7.0	0.932		0.217
	**LM**	60.7 ± 6.9			66.0 ± 11.5			65.8 ± 10.5			67.6 ± 11.4			68.3 ± 7.6			66.6 ± 14.9			0.363
60 min	**HM**	68.7 ± 12.7	0.817		68.5 ± 10.0	0.244		71.6 ± 8.2	0.082		69.6 ± 7.2	0.280		70.6 ± 8.1	0.366		67.6 ± 7.5	0.424		0.789
	**LM**	67.6 ± 12.4			64.5 ± 9.7			67.1 ± 8.8			73.2 ± 11.2			67.6 ± 11.0			71.0 ± 15.6			0.350
**MP VSL (µm/s)**																				
5 min	**HM**	55.4 ± 12.9	0.485	0.466	53.2 ± 12.1	0.883	0.830	53.9 ± 13.2	0.782	0.664	54.5 ± 10.5	0.837	0.468	53.7 ± 13.2	0.625	0.458	54.9 ± 12.0	0.073	0.750	0.991
	**LM**	52.5 ± 11.7		0.472	52.7 ± 13.1		0.641	52.5 ± 12.9		0.865	53.9 ± 10.1		0.887	53.4 ± 10.7		0.930	48.7 ± 11.3		0.777	0.741
30 min	**HM**	50.9 ± 17.6	0.365		52.8 ± 11.7	0.550		53.7 ± 11.4	0.826		52.8 ± 11.8	0.876		54.0 ± 11.6	0.991		53.2 ± 11.3	0.30		0.998
	**LM**	49.0 ± 10.3			50.1 ± 16.7			52.3 ± 14.3			52.3 ± 12.5			54.5 ± 10.5			50.1 ± 12.3			0.823
60 min	**HM**	56.1 ± 13.6	0.777		55.0 ± 11.7	0.836		56.8 ± 11.5	0.804		57.3 ± 12.2	0.206		58.1 ± 12.5	0.181		55.8 ± 11.0	0.351		0.981
	**LM**	54.2 ± 13.5			55.2 ± 11.7			54.6 ± 14.2			53.9 ± 10.7			54.5 ± 11.3			51.7 ± 12.4			0.988
**MP STR (%)**																				
5 min	**HM**	**83.9 ± 11.6***	**0.027**	0.143	80.3 ± 13.0	0.903	0.896	79.4 ± 12.0	0.815	0.680	80.5 ± 9.6	0.865	0.546	79.4 ± 11.8	0.388	0.539	81.0 ± 11.5	0.173	0.643	0.798
	**LM**	**78.1 ± 9.3***		0.879	80.6 ± 13.2		0.272	79.7 ± 13.3		0.949	79.2 ± 11.1		0.768	82.3 ± 11.5		0.821	75.1 ± 14.5		0.961	0.519
30 min	**HM**	76.2 ± 18.3	0.821		79.0 ± 10.6	0.807		76.8 ± 12.6	0.531		78.0 ± 12.2	0.814		76.6 ± 13.2	0.593		79.3 ± 11.5	0.503		0.953
	**LM**	80.0 ± 11.1			75.6 ± 15.4			79.1 ± 11.8			77.9 ± 11.9			80.7 ± 9.9			76.3 ± 13.3			0.757
60 min	**HM**	81.4 ± 9.9	0.656		80.6 ± 10.5	0.270		79.7 ± 11.3	0.497		81.6 ± 11.1	0.055		81.4 ± 11.4	0.492		82.4 ± 9.2	0.105		0.979
	**LM**	79.0 ± 11.4			84.6 ± 8.3			80.5 ± 13.1			76.4 ± 13.9			80.3 ± 9.9			75.2 ± 15.0			0.339

Supplementary Table S3. Continued…

**MP LIN (%)**																				
**5 min**	**HM**	49.3 ± 13.3	0.225	0.435	47.0 ± 16.0	0.705	0.326	44.1 ± 13.7	0.694	0.622	45.7 ± 12.3	0.468	0.677	43.9 ± 13.7	0.510	0.656	45.9 ± 13.5	0.220	0.443	0.791
	**LM**	44.5 ± 10.4		0.771	45.6 ± 14.6		0.747	42.7 ± 10.5		0.756	43.2 ± 11.4		0.885	47.4 ± 11.0		0.872	41.1 ± 12.3		0.425	0.558
**30 min**	**HM**	44.0 ± 15.8	0.567		41.0 ± 10.5	0.915		40.5 ± 12.8	0.195		43.1 ± 11.6	0.681		41.8 ± 12.2	0.101		42.2 ± 11.9	0.910		0.945
	**LM**	46.6 ± 12.0			40.6 ± 12.8			44.4 ± 9.2			41.6 ± 12.1			47.0 ± 15.3			41.6 ± 12.5			0.450
**60 min**	**HM**	46.5 ± 11.5	0.417		44.2 ± 12.4	0.422		42.2 ± 11.6	0.436		43.1 ± 10.0	0.212		44.5 ± 11.5	0.901		**46.6 ± 11.1***	**0.015**		0.803
	**LM**	43.9 ± 10.9			47.6 ± 11.9			45.7 ± 15.1			43.0 ± 13.2			45.1 ± 14.6			**36.1 ± 10.8***			0.297
**MP WOB (%)**																				
**5 min**	**HM**	56.8 ± 9.9	0.553	0.705	56.0 ± 13.1	0.612	0.199	53.5 ± 10.8	0.702	0.587	55.0 ± 10.8	0.718	0.468	53.1 ± 10.4	0.482	0.996	54.6 ± 10.1	0.281	0.345	0.846
	**LM**	55.0 ± 8.2		0.775	54.0 ± 11.7		0.514	52.5 ± 7.8		0.536	53.3 ± 8.5		0.640	55.6 ± 7.8		0.822	53.0 ± 9.9		0.187	0.852
**30 min**	**HM**	54.2 ± 12.1	0.571		50.5 ± 8.1	0.420		50.7 ± 9.9	0.131		52.9 ± 8.1	0.706		52.8 ± 10.4	0.369		51.2 ± 9.2	0.850		0.763
	**LM**	56.2 ± 9.1			52.6 ± 8.3			54.5 ± 6.6			51.9 ± 9.4			56.2 ± 13.1			52.5 ± 10.1			0.567
**60 min**	**HM**	55.6 ± 9.6	0.441		53.2 ± 9.9	0.653		51.4 ± 8.5	0.310		51.7 ± 6.6	0.311		53.0 ± 8.5	0.843		**55.1 ± 8.9***	**0.018**		0.612
	**LM**	53.9 ± 7.2			54.7 ± 9.9			55.0 ± 12.0			54.9 ± 11.1			53.8 ± 13.7			**46.8 ± 8.7***			0.385
**MP ALH (μm)**																				
**5 min**	**HM**	3.1 ± 0.9	0.069	0.339	3.4 ± 1.1	0.660	0.239	3.6 ± 0.9	0.604	0.094	3.6 ± 0.8	0.423	0.448	3.6 ± 1.0	0.633	0.184	3.6 ± 1.0	0.319	0.234	0.415
	**LM**	3.7 ± 0.8		0.365	3.6 ± 1.0		0.251	3.8 ± 1.0		0.615	3.9 ± 1.0		0.678	3.4 ± 0.9		0.279	3.9 ± 1.0		0.066	0.590
**30 min**	**HM**	4.8 ± 6.6	0.713		3.8 ± 0.8	0.817		**4.1 ± 1.0***	**0.017**		3.8 ± 0.8	0.791		4.0 ± 0.9	0.234		4.0 ± 1.0	0.565		0.198
	**LM**	3.3 ± 0.8			3.8 ± 0.7			**3.5 ± 0.6***			3.9 ± 1.0			3.7 ± 1.0			3.8 ± 1.2			0.198
**60 min**	**HM**	3.4 ± 0.7	0.824		3.7 ± 0.8	0.185		4.1 ± 0.8	0.075		3.9 ± 0.5	0.442		3.9 ± 0.7	0.551		**3.6 ± 0.6*****	**<0.001**		0.062
	**LM**	3.5 ± 0.8^{a}			3.4 ± 0.6^{a}			3.6 ± 1.0^{a}			4.1 ± 1.0^{ab}			3.9 ± 0.6^{a}			**4.7 ± 1.0**$^{***\ b}$			**0.001**
**MP BCF (Hz)**																				
**5 min**	**HM**	**25.0 ± 5.0****	**0.007**	0.062	24.0 ± 5.5	0.240	0.407	23.3 ± 4.5	0.063	0.265	23.0 ± 3.6	0.118	0.395	23.2 ± 6.4	0.376	0.372	23.2 ± 4.8	0.078	0.336	0.762
	**LM**	**20.7 ± 4.1****		0.164	21.9 ± 4.8		0.366	20.9 ± 4.2		0.499	21.3 ± 4.6		0.162	22.5 ± 5.0		0.248	20.6 ± 4.8		0.459	0.732
**30 min**	**HM**	21.6 ± 4.9	0.598		22.1 ± 4.3	0.264		21.3 ± 4.2	0.826		21.7 ± 3.5	0.131		21.1 ± 4.6	0.977		21.4 ± 3.6	0.181		0.979
	**LM**	20.7 ± 4.5			20.4 ± 4.9			21.7 ± 3.8			19.8 ± 4.6			21.0 ± 4.5			20.1 ± 8.6			0.882
**60 min**	**HM**	22.5 ± 4.9	0.595		23.4 ± 4.6	0.551		22.0 ± 4.4	0.219		**21.8 ± 3.5***	**0.033**		22.2 ± 4.1	0.120		**22.2 ± 2.9*****	**<0.001**		0.894
	**LM**	21.4 ± 6.2			22.5 ± 3.9			20.1 ± 4.2			**18.2 ± 5.7***			19.8 ± 5.0			**17.9 ± 3.3*****			0.079
**RP VCL (μm/s)**																				
**5 min**	**HM**	141.0 ± 14.0	0.796	0.143	149.6 ± 19.3	0.356	0.226	**143.8 ± 26.9**^{a}	0.715	0.030	152.7 ± 20.6	0.786	0.174	157.1 ± 30.2	0.329	0.441	153.7 ± 20.8	0.220	0.398	0.277
	**LM**	147.0 ± 25.9		0.889	142.8 ± 18.4		0.210	147.1 ± 22.7		0.086	153.1 ± 19.3		0.723	146.9 ± 20.5		0.831	147.5 ± 29.0		0.931	0.933
**30 min**	**HM**	147.1 ± 14.5	0.566		157.9 ± 27.0	0.157		**158.9 ± 26.3**^{ab}	0.125		157.8 ± 22.5	0.549		159.2 ± 21.4	0.222		154.8 ± 22.4	0.755		0.700
	**LM**	143.0 ± 20.3			143.3 ± 22.6			145.2 ± 21.4			152.0 ± 27.2			150.9 ± 27.8			151.5 ± 34.2			0.908
**60 min**	**HM**	151.4 ± 13.2	0.587		164.2 ± 18.2	0.378		**167.8 ± 22.0**^{b}	0.670		169.1 ± 28.3	0.850		168.3 ± 28.0	0.208		163.7 ± 20.2	0.469		0.390
	**LM**	146.8 ± 24.3			156.9 ± 19.6			163.9 ± 25.4			160.1 ± 26.1			153.5 ± 21.9			169.0 ± 57.7			0.737

Supplementary Table S3. Continued…

**RP VAP (μm/s)**																				
**5 min**	**HM**	81.2 ± 13.6	0.438	0.351	80.7 ± 13.3	0.378	0.210	84.9 ± 12.8	0.365	0.156	82.2 ± 13.2	0.983	0.108	**82.4 ± 15.8***	**0.019**	0.065	84.6 ± 12.4	0.114	0.194	0.924
	**LM**	77.4 ± 15.2		0.606	75.8 ± 10.9		0.137	80.7 ± 13.3		0.057	80.0 ± 12.1		0.170	**72.1 ± 7.8***		0.140	78.2 ± 15.3		0.915	0.600
**30 min**	**HM**	85.5 ± 12.6	0.065		81.3 ± 14.4	0.441		84.6 ± 11.9	0.399		82.9 ± 11.5	0.682		86.4 ± 12.7	0.180		83.4 ± 11.6	0.334		0.878
	**LM**	75.7 ± 11.4			77.3 ± 10.7			80.6 ± 15.0			85.0 ± 15.2			82.5 ± 20.2			77.7 ± 18.6			0.724
**60 min**	**HM**	88.4 ± 13.7	0.316		90.1 ± 17.2	0.803		92.1 ± 10.7	0.890		91.1 ± 12.2	0.849		94.3 ± 15.4	0.227		91.5 ± 13.9	0.469		0.925
	**LM**	82.0 ± 14.8			88.4 ± 15.6			92.9 ± 15.6			92.2 ± 16.6			85.9 ± 15.7			88.7 ± 28.5			0.751
**RP VSL (μm/s)**																				
**5 min**	**HM**	75.0 ± 14.2	0.410	0.565	73.4 ± 13.4	0.380	0.277	78.3 ± 13.2	0.331	0.295	74.2 ± 12.5	0.851	0.180	**73.4 ± 14.3**$^{*\,a}$	**0.010**	0.045	76.6 ± 12.2	0.087	0.283	0.850
	**LM**	69.3 ± 15.4		0.791	69.1 ± 11.5		0.065	73.6 ± 14.5		0.111	72.2 ± 11.9		0.185	**64.3 ± 6.6***		0.125	70.8 ± 14.6		0.482	0.588
**30 min**	**HM**	77.7 ± 14.2	0.132		72.6 ± 13.5	0.395		75.7 ± 11.9	0.570		74.1 ± 10.6	0.547		**77.1 ± 12.0**^{ab}	0.222		75.7 ± 11.6	0.261		0.879
	**LM**	69.1 ± 11.7			68.6 ± 8.7			72.9 ± 15.2			77.1 ± 15.0			74.8 ± 19.3			69.4 ± 17.3			0.690
**60 min**	**HM**	80.7 ± 13.6	0.201		81.0 ± 17.1	0.867		82.4 ± 9.7	0.744		81.1 ± 10.8	0.626		**85.2 ± 14.7**^{b}	0.223		82.7 ± 14.0	0.694		0.950
	**LM**	73.0 ± 13.4			79.9 ± 15.0			83.9 ± 14.5			83.8 ± 16.1			77.3 ± 14.0			79.3 ± 25.1			0.686
**RP STR (%)**																				
**5 min**	**HM**	**91.9 ± 3.1***	**0.043**	0.507	90.6 ± 2.8	0.985	0.357	**91.9 ± 3.5**^{a}	0.389	0.019	90.0 ± 4.1	0.917	0.628	88.9 ± 4.2	0.715	0.538	90.4 ± 3.4	0.713	0.928	0.090
	**LM**	**89.3 ± 3.8***		0.324	90.5 ± 3.4		0.389	90.7 ± 4.7		0.922	89.9 ± 2.9		0.835	89.1 ± 2.1		0.461	89.9 ± 3.2		0.842	0.860
**30 min**	**HM**	90.6 ± 3.7	0.760		89.1 ± 2.5	0.739		**89.2 ± 2.9**^{b}	0.391		89.2 ± 2.5	0.289		89.0 ± 3.4	0.251		90.5 ± 2.1	0.220		0.376
	**LM**	91.1 ± 2.9			88.7 ± 3.6			90.1 ± 3.2			90.4 ± 3.2			90.4 ± 3.0			89.0 ± 4.3			0.562
**60 min**	**HM**	91.0 ± 2.4	0.105		89.5 ± 3.4	0.650		**89.3 ± 2.9**^{b}	0.393		88.9 ± 2.7	0.190		90.1 ± 2.3	0.945		90.1 ± 3.2	0.718		0.529
	**LM**	88.9 ± 3.2			90.1 ± 2.6			90.3 ± 2.7			90.7 ± 3.6			90.0 ± 2.1			89.5 ± 4.2			0.856
**RP LIN (%)**																				
**5 min**	**HM**	54.9 ± 9.8	0.057	0.997	50.6 ± 10.7	0.859	0.612	57.4 ± 15.2	0.199	0.097	50.6 ± 9.5	0.786	0.671	48.8 ± 10.8	0.591	0.513	51.8 ± 10.8	0.561	0.924	0.217
	**LM**	48.7 ± 12.2		0.898	49.8 ± 10.3		0.686	51.1 ± 11.2		0.959	48.4 ± 7.7		0.398	45.1 ± 5.9		0.089	49.4 ± 11.0		0.832	0.781
**30 min**	**HM**	54.9 ± 12.0	0.558		47.7 ± 8.5	0.515		49.6 ± 8.5	0.676		48.1 ± 7.1	0.354		50.0 ± 8.7	0.589		50.8 ± 8.3	0.252		0.312
	**LM**	50.1 ± 12.9			49.9 ± 8.1			50.8 ± 7.9			52.9 ± 11.8			50.7 ± 8.5			46.9 ± 8.6			0.810
**60 min**	**HM**	54.6 ± 9.9	0.503		51.5 ± 12.0	0.756		50.6 ± 8.8	0.199		49.2 ± 6.1	0.345		52.6 ± 9.9	0.951		52.1 ± 10.3	0.290		0.791
	**LM**	51.4 ± 12.0			52.9 ± 8.6			51.8 ± 6.7			54.2 ± 12.7			51.2 ± 4.3			48.0 ± 3.0			0.786
**RP WOB (%)**																				
**5 min**	**HM**	59.2 ± 9.0	0.086	0.977	55.4 ± 10.4	0.818	0.614	61.8 ± 14.2	0.193	0.153	55.7 ± 9.0	0.722	0.761	54.3 ± 10.1	0.576	0.520	56.8 ± 10.3	0.518	0.875	0.296
	**LM**	54.1 ± 11.3		0.812	54.5 ± 9.7		0.629	55.9 ± 9.9		0.911	53.4 ± 7.1		0.335	50.3 ± 5.7		0.066	54.3 ± 10.4		0.881	0.786
**30 min**	**HM**	59.9 ± 10.8	0.412		53.1 ± 8.1	0.405		55.2 ± 8.1	0.751		53.6 ± 6.7	0.407		55.7 ± 8.1	0.857		55.7 ± 8.1	0.271		0.318
	**LM**	54.6 ± 12.5			55.9 ± 8.5			56.1 ± 7.0			57.9 ± 10.8			55.8 ± 7.8			52.3 ± 7.2			0.788
**60 min**	**HM**	59.6 ± 9.5	0.651		56.8 ± 11.2	0.755		56.3 ± 8.3	0.369		55.1 ± 5.6	0.529		58.0 ± 10.1	0.951		57.3 ± 9.5	0.277		0.862
	**LM**	57.4 ± 12.3			58.2 ± 8.2			57.1 ± 5.9			59.3 ± 12.0			56.6 ± 4.1			53.5 ± 2.2			0.677

Supplementary Table S3. Continued…

**RP ALH (μm)**																				
**5 min**	**HM**	3.3 ± 0.7	0.385	0.431	4.9 ± 4.3	0.307	0.675	**3.4 ± 1.1**^{a}	0.165	**0.018**	**3.7 ± 0.8**^{a}	0.213	**0.026**	4.1 ± 1.3	0.677	0.983	3.7 ± 0.9	0.793	0.313	0.179
	**LM**	4.0 ± 0.9		0.401	3.5 ± 1.0		0.556	3.9 ± 0.9		0.018	4.0 ± 1.0		0.989	3.9 ± 0.9		0.883	3.8 ± 1.2		0.733	0.832
**30 min**	**HM**	3.6 ± 0.8	0.725		4.1 ± 1.0	0.582		**4.1 ± 0.8**^{b}	0.250		**4.2 ± 0.9**^{ab}	0.573		4.1 ± 0.9	0.636		4.0 ± 0.8	0.780		0.556
	**LM**	3.5 ± 0.8			3.9 ± 1.0			3.7 ± 0.7			3.9 ± 1.1			4.0 ± 1.0			3.9 ± 1.0			0.886
**60 min**	**HM**	3.6 ± 0.7	0.987		4.1 ± 0.9	0.489		**4.3 ± 0.9**^{b}	0.512		**4.5 ± 0.8**^{b}	0.975		4.1 ± 0.8	0.903		4.3 ± 1.2	0.690		0.197
	**LM**	3.6 ± 1.0			3.8 ± 0.8			4.2 ± 0.8			4.0 ± 1.2			4.1 ± 0.7			4.2 ± 1.2			0.709
**RP BCF (Hz)**																				
**5 min**	**HM**	**27.1 ± 5.0*****	**<0.001**	0.452	25.2 ± 3.2	0.745	0.289	**25.7 ± 3.9**^{a}	0.059	**0.039**	**26.6 ± 4.7**^{a}	0.217	**0.010**	23.5 ± 4.6	0.817	0.342	25.3 ± 5.4	0.18	0.324	0.253
	**LM**	**16.3 ± 9.0**$^{***\ a\ a}$		**0.040**	26.8 ± 6.3^{b}		0.102	22.2 ± 6.3^{b}		0.799	23.3 ± 4.4^{b}		0.383	23.1 ± 4.4^{b}		0.687	22.9 ± 3.7^{b}		0.724	**0.020**
**30 min**	**HM**	25.5 ± 6.4	0.847		23.0 ± 4.0	0.959		**22.8 ± 3.3**^{b}	0.609		**22.7 ± 3.0**^{b}	0.606		23.3 ± 4.0	0.444		23.0 ± 3.5	0.269		0.450
	**LM**	**24.9 ± 6.4**^{b}			21.2 ± 8.0			23.5 ± 4.6			23.2 ± 2.6			22.2 ± 3.7			22.3 ± 6.3			0.450
**60 min**	**HM**	24.5 ± 4.6	0.630		23.8 ± 4.0	0.314		**23.1 ± 4.0**^{ab}	0.630		**23.2 ± 3.0b**	0.207		**25.3 ± 4.2***	**0.038**		23.4 ± 5.2	0.320		0.689
	**LM**	**25.8 ± 7.1**^{ab}			25.5 ± 3.8			22.2 ± 5.9			21.2 ± 4.4			**21.7 ± 2.8***			21.1 ± 4.8			0.165

ALH, average path velocity; BCF, beat cross frequency; CAP, capacitating-HTF; HTF, human tubal fluid; HM, high motile subpopulation; LIN, linearity; LM, low motile subpopulation; MP, medium-progressive; min, minutes; NP, non-progressive; PRL, prolactin; Prog, progressive; RP, rapid-progressive; SD, standard deviation; SP *p*, t-test between subpopulations; STR, straightness; TP *p*, t test between time points; VAP, average path velocity; VCL, curvilinear velocity; VSL, straight-line velocity; WOB, wobble. Values labelled in bold with an asterisks in the same column were significantly different between the HM and LM subpopulations for individual media and SCA parameters **(*p <0.05, **p <0.01 and ***p <0.001)**. Values labelled in bold and red with an asterisks in the same column were significantly different between the 5 and 30 minutes for individual subpopulations and media **(*p <0.05 and **p <0.01).** Student's t-test was used or the Mann-Whitney test when normal distribution was absent. $^{a,\ b,c,d}$ Values labelled with different superscript letters in the same row were significantly different between the various media for individual subpopulations and SCA parameters. One-way ANOVA was used for parametric distributions or Kruskal-Wallis test for non-parametric distributions.

Supplementary Table S4 - Percentage hyperactivation in both low motile and high motile sperm subpopulations after treatment with HTF, CAP, HD-C, 1.98 µM progesterone, 3.96 µM progesterone, 19.8 µM progesterone and 11 mM myo-inositol at 5, 15, 30, 45 and 60 minutes (mean±SD) (n=20).

	**HTF**		**CAP**		**HD-C**		**1.98 µM PRG**		**3.96 µM PRG**		**19.8 µM PRG**		**11 mM MYO**		
	**Mean ± SD**	**SP *p***	**Mean ± SD**	**SP *p***	**Mean ± SD**	**SP *p***	**Mean ± SD**	**SP *p***	**Mean ± SD**	**SP *p***	**Mean ± SD**	**SP *p***	**Mean ± SD**	**SP *p***	**ANOVA**
**5 minutes**															
**HM**	18.0 ± 11.5^{a}	0.081	29.8 ± 17.1^{ab}	0.133	42.4 ± 20.1^{b}	0.163	29.8 ± 17.8^{ab}	0.185	37.9 ± 18.8^{b}	0.210	35.67 ± 17.8^{b}	0.095	36.1 ± 17.0$^{*\,b}$	**0.022**	**0.005**
**LM**	10.8 ± 9.5^{a}		22.6 ± 14.7^{ab}		33.5 ± 19.3^{b}		23.2 ± 16.9^{ab}		30.2 ± 19.5^{b}		26.0 ± 18.0^{ab}		23.9 ± 16.1$^{*\,ab}$		**0.009**
**15 minutes**															
**HM**	25.0 ± 13.3***	**<0.001**	35.4 ± 17.2	0.085	43.1 ± 16.1	0.217	34.5 ± 16.7	0.178	41.0 ± 16.7*	**0.022**	37.1 ± 16.6	0.060	35.6 ± 16.7	0.149	0.067
**LM**	8.8 ± 5.8$^{***\,a}$		26.2 ± 15.8^{b}		35.7 ± 21.1^{b}		26.2 ± 21.4^{b}		27.7 ± 18.7$^{*\,b}$		27.0 ± 21.7^{b}		27.8 ± 16.9^{b}		**0.003**
**30 minutes**															
**HM**	24.1 ± 11.4***	**<0.001**	35.2 ± 17.9	0.082	35.9 ± 16.1	0.230	29.4 ± 16.0	0.404	34.7 ± 16.8	0.091	30.8 ± 17.0	0.151	27.6 ± 13.7	0.414	0.245
**LM**	8.64 ± 8.5***		25.6 ± 16.3		28.5 ± 21.7		24.5 ± 20.5		25.1 ± 18.2		23.5 ± 20.0		23.3 ± 18.9		0.100
**45 minutes**															
**HM**	17.2 ± 9.9***	**<0.001**	27.9 ± 12.5	0.079	27.1 ± 14.9	0.605	25.1 ± 16.7	0.122	30.4 ± 15.2	0.085	23.9 ± 12.2	0.176	23.8 ± 11.9	0.599	0.172
**LM**	4.0 ± 6.6$^{***\,a}$		20.2 ± 14.6^{b}		24.4 ± 17.7^{b}		17.7 ± 16.9^{b}		21.7 ± 16.2^{b}		19.4 ± 18.8^{b}		21.2 ± 18.1^{b}		**0.008**
**60 minutes**															
**HM**	13.8 ± 9.8**	**0.005**	21.0 ± 14.8	0.212	21.7 ± 12.7	0.568	21.1 ± 12.3	0.098	25.5 ± 14.7*	**0.021**	20.8 ± 12.1	0.050	20.1 ± 10.2	0.133	0.304
**LM**	3.0 ± 7.0$^{**\,a}$		16.0 ± 15.1^{b}		19.4 ± 13.3^{b}		15.46 ± 17.5^{b}		14.8 ± 13.9$^{*\,b}$		12.7 ± 12.9^{b}		14.7 ± 12.0^{b}		**0.024**

CAP, capacitating-HTF; HD-C, HD capacitating medium; HM, high motile; HTF, human tubal fluid; LM, low motile; min, minutes; MYO, myo-inositol; PRG, progesterone; SD, standard deviation and SP *p*, t test between subpopulations. $^{a,\,b,c,d}$ Values labelled with different superscript letters in the same row were significantly different between the various media for individual subpopulations and time points. One-way ANOVA for parametric distributions or Kruskal-Wallis test for non-parametric distributions was used. Values labelled with an asterisks in the same column were significantly different between the HM and LM subpopulations for individual media and time points (**p* <0.05, ***p* <0.01 and ****p* <0.001). Student's t-test was used or the Mann-Whitney test when normal distribution was void.

Supplementary Table S5 - Percentage hyperactivation in both low motile and high motile sperm subpopulations after treatment with HTF, CAP, 20 nM, 100 nM and 1 µM dopamine (mean±SD) (n=20).

	**HTF**		**CAP**		**20 nM DOPA**		**100 nM DOPA**		**1 µM DOPA**		
	**Mean ± SD**	**SP *p***	**Mean ± SD**	**SP *p***	**Mean ± SD**	**SP *p***	**Mean ± SD**	**SP *p***	**Mean ± SD**	**SP *p***	**ANOVA**
**5 minutes**											
**HM**	11.0 ± 15.0^{a}	0.544	19.7 ± 18.0^{a}	0.054	35.7 ± 21.9$^{**\,b}$	**0.004**	31.3 ± 15.4^{b}	0.158	35.2 ± 21.0$^{*\,b}$	**0.028**	< **0.001**
**LM**	6.8 ± 7.4^{a}		11.9 ± 12.2^{ac}		19.0 ± 15.0$^{**\,bc}$		24.0 ± 16.9^{b}		22.1 ± 15.1$^{*\,b}$		< **0.001**
**15 minutes**											
**HM**	17.8 ± 13.7$^{**\,a}$	**0.003**	29.4 ± 17.3$^{**\,b}$	**0.006**	35.1 ± 20.4$^{**\,b}$	**0.009**	31.3 ± 12.5$^{*\,b}$	**0.028**	39.5 ± 18.2$^{*\,b}$	**0.015**	**0.001**
**LM**	6.9 ± 6.8$^{**\,a}$		15.7 ± 11.9$^{**\,b}$		18.9 ± 12.6$^{**\,bc}$		21.6 ± 14.2$^{*\,bc}$		26.7 ± 13.4$^{*\,c}$		< **0.001**
**30 minutes**											
**HM**	23.1 ± 14.0***	<**0.001**	33.6 ± 19.4***	<**0.001**	33.6 ± 18.4*	**0.010**	33.6 ± 15.6*	**0.011**	37.9 ± 19.2	0.060	0.105
**LM**	6.6 ± 9.3$^{***\,a}$		13.7 ± 12.0$^{***\,ab}$		20.5 ± 11.7$^{*\,bc}$		21.4 ± 13.3$^{*\,bc}$		24.5 ± 15.3^{c}		< **0.001**
**45 minutes**											
**HM**	12.0 ± 13.1^{a}	0.146	24.8 ± 15.6$^{**\,ab}$	**0.002**	20.8 ± 16.0^{ab}	0.329	24.2 ± 16.8^{b}	0.119	31.8 ± 16.3^{b}	0.056	**0.003**
**LM**	5.9 ± 8.0^{a}		10.3 ± 10.9$^{**\,ab}$		16.7 ± 12.1^{bc}		18.2 ± 15.8^{bc}		21.7 ± 16.1^{c}		**0.001**
**60 minutes**											
**HM**	9.7 ± 12.1^{a}	0.078	22.2 ± 12.1$^{**\,ab}$	**0.001**	21.2 ± 16.0^{b}	0.078	18.0 ± 17.1^{ab}	0.430	23.4 ± 18.0^{b}	0.067	**0.041**
**LM**	4.7 ± 8.9		9.6 ± 10.7**		14.3 ± 12.0		14.4 ± 14.9		13.0 ± 12.5		0.056

CAP, capacitating-HTF; DOPA, dopamine; HM, high motile; HTF, human tubal fluid; LM, low motile; min, minutes; SD, standard deviation and SP *p*, t test between subpopulations. [a, b,c,d] Values labelled with different superscript letters in the same row were significantly different between the various media for individual subpopulations and time points. One-way ANOVA for parametric distributions or Kruskal-Wallis test for non-parametric distributions was used. Values labelled with an asterisks in the same column were significantly different between the HM and LM subpopulations for individual media and time points (**p* <0.05, ***p* <0.01 and ****p* <0.001). Student's t-test was used or the Mann-Whitney test when normal distribution was void.

Supplementary Table S6 - Percentage hyperactivation in both low motile and high motile sperm subpopulations after treatment with HTF, CAP, 50 ng/mL, 100 ng/mL, 250 ng/mL and 500 ng/mL prolactin at 5, 15, 30, 45 and 60 minutes (mean±SD) (n=20).

	HTF		CAP		50 ng/mL PRL		100 ng/mL PRL		250 ng/mL PRL		500 ng/mL PRL		
	**Mean ± SD**	**SP *p***	**Mean ± SD**	**SP *p***	**Mean ± SD**	**SP *p***	**Mean ± SD**	**SP *p***	**Mean ± SD**	**SP *p***	**Mean ± SD**	**SP *p***	**ANOVA**
**5 minutes**													
**HM**	$18.9 \pm 13.7^{***}$	**<0.001**	$29.6 \pm 17.4^{**}$	**0.003**	26.4 ± 18.1	0.451	29.7 ± 18.3	0.354	28.0 ± 19.1	0.132	**$34.0 \pm 18.6^{*}$**	**0.040**	0.091
**LM**	$7.9 \pm 9.5^{***\,a}$		$15.0 \pm 13.8^{**\,ab}$		22.4 ± 18.1^{b}		22.0 ± 17.6^{b}		18.9 ± 18.7^{b}		**$23.6 \pm 15.3^{*\,b}$**		**0.005**
**15 minutes**													
**HM**	$22.3 \pm 8.2^{**\,a}$	**0.001**	$34.5 \pm 14.8^{**\,b}$	**0.003**	36.8 ± 19.7^{b}	0.155	32.2 ± 18.2^{b}	0.967	33.3 ± 18.9^{b}	0.302	33.9 ± 17.0^{b}	0.318	**0.029**
**LM**	$11.5 \pm 11.6^{**\,a}$		$21.1 \pm 14.5^{**\,b}$		28.9 ± 18.0^{b}		32.5 ± 21.0^{b}		26.7 ± 20.1^{b}		29.1 ± 16.4^{b}		**0.001**
**30 minutes**													
**HM**	$21.3 \pm 10.7^{***\,a}$	**<0.001**	$35.3 \pm 17.7^{**\,ab}$	**0.001**	$38.2 \pm 18.5^{**\,b}$	**0.009**	33.6 ± 18.2^{b}	0.380	34.4 ± 21.6^{ab}	0.410	32.9 ± 17.5^{b}	0.155	**0.026**
**LM**	$9.5 \pm 10.1^{***\,a}$		$19.6 \pm 13.8^{**\,b}$		$24.9 \pm 15.6^{**\,bc}$		28.8 ± 19.2^{bc}		29.7 ± 17.6^{c}		25.4 ± 18.3^{bc}		**<0.001**
**45 minutes**													
**HM**	$16.1 \pm 10.7^{*\,a}$	**0.014**	$26.5 \pm 15.9^{*\,b}$	**0.012**	$32.5 \pm 16.8^{*\,b}$	**0.017**	29.0 ± 14.4^{b}	0.642	$35.1 \pm 18.0^{*\,b}$	**0.017**	29.4 ± 15.3^{b}	0.188	**0.001**
**LM**	$8.7 \pm 10.3^{*\,a}$		$16.1 \pm 11.8^{*\,ab}$		$21.3 \pm 14.8^{*\,b}$		25.6 ± 17.7^{b}		$23.9 \pm 12.9^{*\,b}$		23.6 ± 14.6^{b}		**<0.001**
**60 minutes**													
**HM**	$17.7 \pm 11.9^{**\,a}$	**0.001**	$25.4 \pm 15.4^{*\,ab}$	**0.015**	$31.0 \pm 16.6^{*\,ab}$	**0.010**	26.0 ± 12.9^{ab}	0.692	$30.7 \pm 19.6^{**\,b}$	**0.008**	$28.8 \pm 18.0^{*\,ab}$	**0.016**	**0.049**
**LM**	$7.0 \pm 9.5^{**\,a}$		$14.8 \pm 10.8^{*\,b}$		$19.8 \pm 12.3^{*\,b}$		24.4 ± 15.0^{b}		$17.1 \pm 14.6^{**\,b}$		$17.9 \pm 11.9^{*\,b}$		**<0.001**

CAP, capacitating-HTF; HM, high motile; HTF, human tubal fluid; LM, low motile; min, minutes; PRL, prolactin; SD, standard deviation and SP *p*, t test between subpopulations. [a, b,c,d] Values labelled with different superscript letters in the same row were significantly different between the various media for individual subpopulations and time points. One-way ANOVA for parametric distributions or Kruskal-Wallis test for non-parametric distributions was used. Values labelled with an asterisks in the same column were significantly different between the HM and LM subpopulations for individual media and time points (**p* <0.05, ***p* <0.01 and ****p* <0.001). Student's t-test was used or the Mann-Whitney test when normal distribution was void.

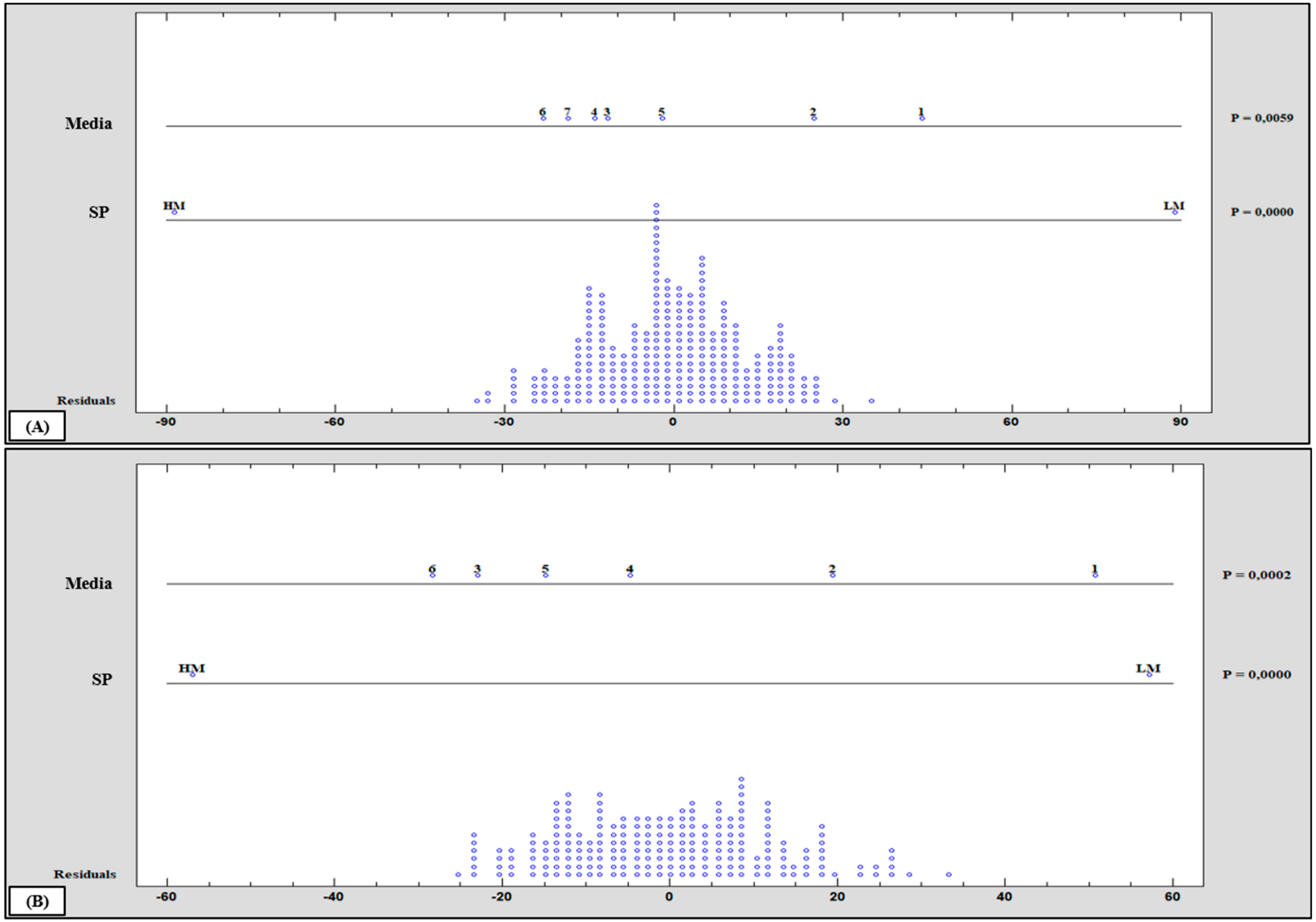

Supplementary Figure S1. Multifactorial graphical ANOVA of the pooled data from the high motile (HM) and low motile (LM) sperm subpopulations percentage reactive oxygen species (ROS) positive spermatozoa. **(A)** Multifactorial graphical ANOVA displaying the significant interactions of subpopulations and media on ROS percentages after treatment with HTF (1), CAP (2), HD-C (3), 1.98 µM PRG (4), 3.96 µM PRG (5), 19.8 µM PRG (6) and 11 mM MYO (7). **(B)** Multifactorial graphical ANOVA displaying the significant interactions of subpopulations and media on ROS percentages after treatment with HTF (1), CAP (2), 50 ng/mL PRL (3) 100 ng/mL PRL (4), 250 ng/mL PRL (5) and 500 ng/mL PRL (6) The Graphical ANOVA plot shows the effects of each factor scaled so that they can be compared to the variability of the residuals. For each factor, the deviations of the adjusted level means from the estimated grand mean are displayed. Any factor that shows considerably larger variability than the residuals is likely to be an important factor. Student-Newman-Keuls was used for the post hoc test. ANOVA, analysis of variance; CAP, capacitating-HTF; HD-C, HD capacitation medium; HM, high motile subpopulation; HTF, human tubal fluid; LM, low motile subpopulation; MYO, myo-inositol; PRG, progesterone; PRL, prolactin.; ROS, positive reactive oxygen species

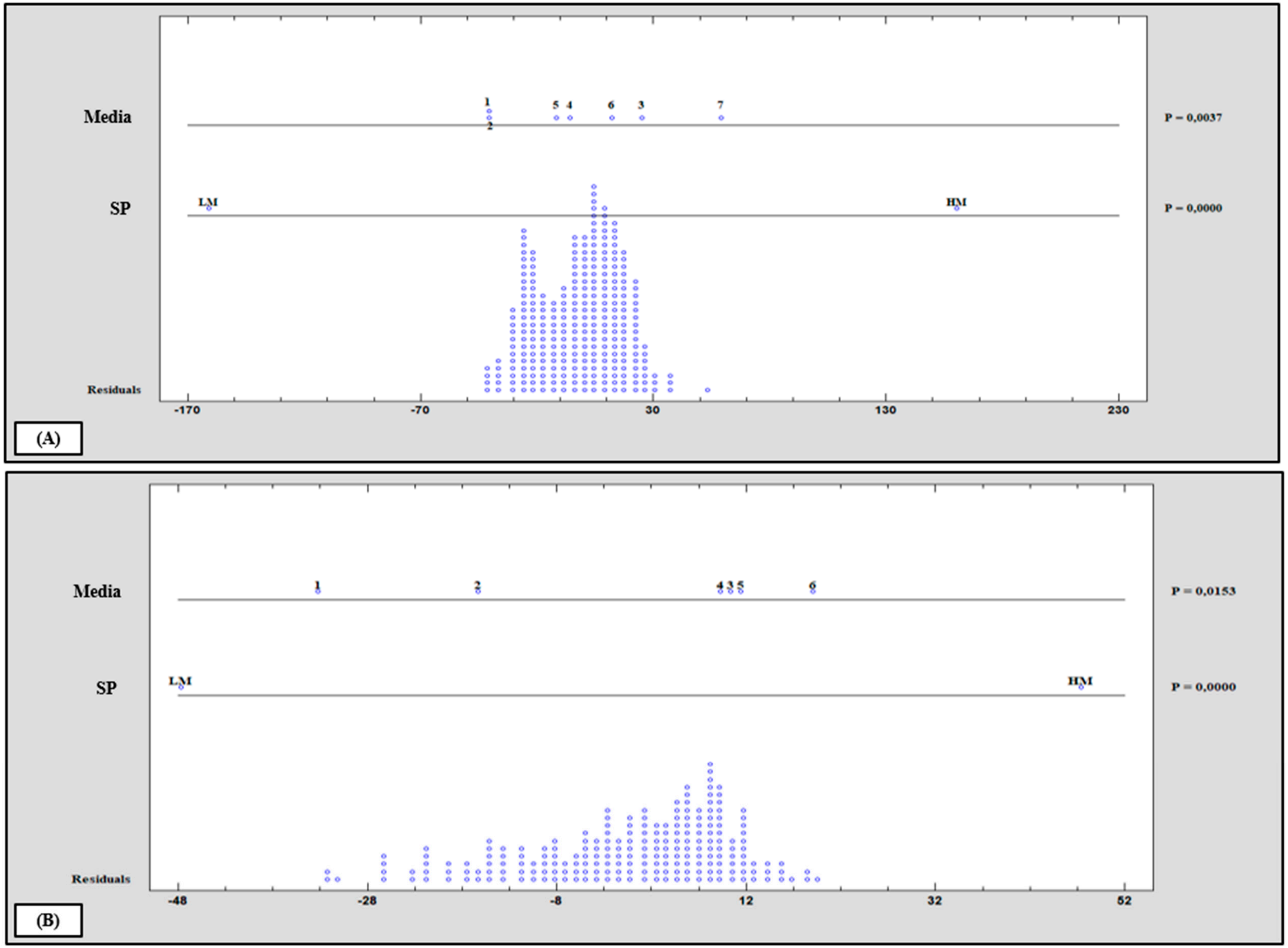

Supplementary Figure S2. Multifactorial graphical ANOVA of the pooled data from the high motile (HM) and low motile (LM) sperm subpopulations percentage intact mitochondrial membrane potential (MMP). **(A)** Multifactorial graphical ANOVA displaying the significant interactions of subpopulations and media on MMP intactness percentages after treatment with HTF (1), CAP (2), HD-C (3), 1.98 µM PRG (4), 3.96 µM PRG (5), 19.8 µM PRG (6) and 11 mM MYO (7). **(B)** Multifactorial graphical ANOVA displaying the significant interactions of subpopulations and media on MMP intactness percentages after treatment with HTF (1), CAP (2), 50 ng/mL PRL (3) 100 ng/mL PRL (4), 250 ng/mL PRL (5) and 500 ng/mL PRL (6) The Graphical ANOVA plot shows the effects of each factor scaled so that they can be compared to the variability of the residuals. For each factor, the deviations of the adjusted level means from the estimated grand mean are displayed. Any factor that shows considerably larger variability than the residuals is likely to be an important factor. Student-Newman-Keuls was used for the post hoc test. ANOVA, analysis of variance; CAP, capacitating-HTF; HD-C, HD capacitation medium; HM, high motile subpopulation; HTF, human tubal fluid; LM, low motile subpopulation; MMP, mitochondrial membrane intactness; MYO, myo-inositol; PRG, progesterone; PRL, prolactin and SP, subpopulations.

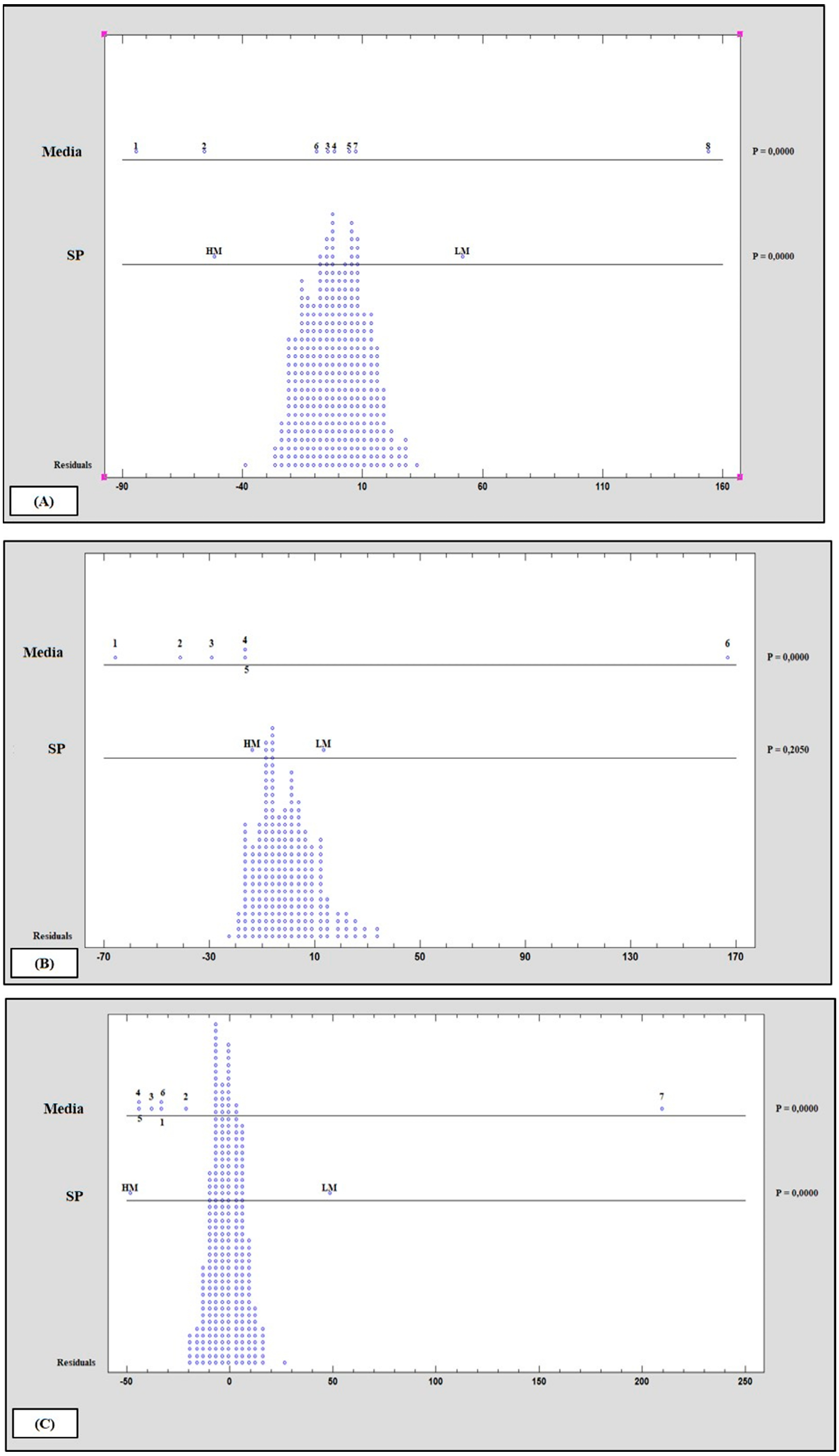

Supplementary Figure S3. Multifactorial graphical ANOVA of the pooled data from the high motile (HM) and low motile (LM) sperm subpopulations percentage acrosome reaction (AR). **(A)** Multifactorial graphical ANOVA displaying the significant interactions of subpopulations and media on AR percentages after treatment with HTF (1), CAP (2), HD-C (3), 1.98 µM PRG (4), 3.96 µM PRG (5), 19.8 µM PRG (6), 11 mM MYO (7) and Ca-ionophore (8). **(B)** Multifactorial graphical ANOVA displaying the significant interactions of subpopulations and media on AR percentages after treatment with HTF (1), CAP (2), 20 nM DOPA (3) 100 nM DOPA (4), 1 µM DOPA (5) and Ca-ionophore (6). **(C)** Multifactorial graphical ANOVA displaying the significant interactions of subpopulations and media on AR percentages after treatment with HTF (1), CAP (2), 50 ng/mL PRL (3) 100 ng/mL PRL (4), 250 ng/mL PRL (5), 500 ng/mL PRL (6) and Ca-ionophore (7). The Graphical ANOVA plot shows the effects of each factor scaled so that they can be compared to the variability of the residuals. For each factor, the deviations of the adjusted level means from the estimated grand mean are displayed. Any factor that shows considerably larger variability than the residuals is likely to be an important factor. Student-Newman-Keuls was used for the post hoc test. ANOVA, analysis of variance; AR, acrosome reaction; CAP, capacitating-HTF; Ca-ionophore, calcium ionophore; DOPA, dopamine; HD-C, HD capacitation medium; HM, high motile subpopulation; HTF, human tubal fluid; LM, low motile subpopulation; MYO, myo-inositol; PRG, progesterone; PRL, prolactin and SP, subpopulations.
